# Deep Learning and Machine Learning Algorithms for Retinal Image Analysis in Neurodegenerative Disease: Systematic Review of Datasets and Models

**DOI:** 10.1167/tvst.13.2.16

**Published:** 2024-02-21

**Authors:** Tyler Bahr, Truong A. Vu, Jared J. Tuttle, Raymond Iezzi

**Affiliations:** 1 Mayo Clinic, Department of Ophthalmology, Rochester, MN, USA; 2 University of the Incarnate Word, School of Osteopathic Medicine, San Antonio, TX, USA; 3 University of Texas Health Science Center at San Antonio, Joe R. and Teresa Lozano Long School of Medicine, San Antonio, TX, USA

**Keywords:** convolutional neural network, deep learning, machine learning, neurodegenerative disease, alzheimer's disease (AD), parkinson's disease (PD), huntington's disease (HD), amyotrophic lateral sclerosis (ALS), dementia, mild cognitive impairment, optical coherence tomography (OCT), retinal imaging, fundus

## Abstract

**Purpose:**

Retinal images contain rich biomarker information for neurodegenerative disease. Recently, deep learning models have been used for automated neurodegenerative disease diagnosis and risk prediction using retinal images with good results.

**Methods:**

In this review, we systematically report studies with datasets of retinal images from patients with neurodegenerative diseases, including Alzheimer's disease, Huntington's disease, Parkinson's disease, amyotrophic lateral sclerosis, and others. We also review and characterize the models in the current literature which have been used for classification, regression, or segmentation problems using retinal images in patients with neurodegenerative diseases.

**Results:**

Our review found several existing datasets and models with various imaging modalities primarily in patients with Alzheimer's disease, with most datasets on the order of tens to a few hundred images. We found limited data available for the other neurodegenerative diseases. Although cross-sectional imaging data for Alzheimer's disease is becoming more abundant, datasets with longitudinal imaging of any disease are lacking.

**Conclusions:**

The use of bilateral and multimodal imaging together with metadata seems to improve model performance, thus multimodal bilateral image datasets with patient metadata are needed. We identified several deep learning tools that have been useful in this context including feature extraction algorithms specifically for retinal images, retinal image preprocessing techniques, transfer learning, feature fusion, and attention mapping. Importantly, we also consider the limitations common to these models in real-world clinical applications.

**Translational Relevance:**

This systematic review evaluates the deep learning models and retinal features relevant in the evaluation of retinal images of patients with neurodegenerative disease.

## Introduction

The retina is the only neural tissue in the human body that can be directly visualized noninvasively. Findings from retinal imaging can be informative regarding the health of the brain; many abnormalities in retinal imaging have been linked with cerebral pathology.^1^

In the past decade, image analysis has been revolutionized by convolutional neural networks and other promising emerging technologies for automated image analysis. These technologies undergoing further development may possibly augment the diagnostic capabilities of several ophthalmologic imaging modalities. Machine learning algorithms may assist us in detecting information in retinal images that may not be readily apparent without computational algorithms.^2^ Several groups have been using machine learning algorithms to determine if systemic patient health information can be gleaned from retinal images. Such algorithms have demonstrated good accuracy at predicting quantitative variables, such as coronary artery calcium or serum creatinine, and also qualitative variables, such as smoking status or biological sex using retinal fundus images alone.^3^ It is difficult to identify which features in the retinal images are used by the machine learning algorithms to glean this information. However, there may be more information contained in retinal images than was previously known, and it may be necessary to apply computational algorithms, such as machine learning to establish this.^4^

The retina is known to exhibit many of the classic pathologic features of neurodegenerative disease. Multiple postmortem pathology studies have identified beta-amyloid plaques and neurofibrillary tangles in the retina of patients with varying stages of Alzheimer's disease (AD).^5^^–^^8^ In patients with Parkinson's disease (PD), pathology studies have found lower levels of dopamine in the retina.^9^^,^^10^ The neurodegeneration that accompanies Huntington's disease (HD) and amyotrophic lateral sclerosis (ALS) may appear in the retina as well.^11^^,^^12^ Case-control comparisons suggest that patients with mild cognitive impairment (MCI) or unspecified dementia (D-US) also exhibit retinal thinning.^13^^,^^14^

Given the known correlation between retinal health and neurodegenerative disease, there is good potential that deep learning algorithms might be able to ascertain information regarding cerebral disease from retinal images.^15^ Indeed, a growing amount of literature has documented correlations between the progression of neurodegenerative disease and physician-observable retinal findings, such as retinal arteriolar and venular caliber, vessel tortuosity, retinal layer thickness, and optic disc morphology.^16^ Future research will likely focus on determining what information is contained within optical coherence tomography (OCT), OCT-angiography (OCT-A), and color fundus images.^15^ Such studies will also need to consider what information is not able to be obtained from retinal imaging.

OCT, OCT-A, and fundus imaging allow for detailed quantitative and qualitative analysis of retinal features. OCT uses the reflectivity of light to micro-image the anatomy of the retina and optic disk. The peripapillary retinal nerve fiber layer (pRNFL) and macular ganglion cell layer and inner plexiform layer (mGCIPL) are especially implicated in neurodegenerative states, whereas other markers, such as macular volume and choroidal thickness, have also been studied. OCT-A works by comparing retinal layers across time as blood flows through the capillaries. OCT-A captures information regarding retinal vasculature, including microvascular density, branching complexity, and flow density. Fundus imaging allows for the direct visualization of the macula, optic disk, and retinal vasculature. Vessel tortuosity and branching complexity have been identified as helpful biomarkers and other retinal features can be directly visualized through the use of fluorescence imaging. Each imaging modality provides a host of information that has revealed retinal manifestations of neurodegenerative disease.

Presently, it seems that current research using retinal imaging has only scratched the surface of the information which deep learning algorithms might provide, but there are also significant limitations that have yet to be addressed. Many non-neurologic diseases can have retinal manifestations indistinguishable from the features reportedly used by current machine learning models to distinguish between healthy eyes and ones with neurodegenerative disease. It will be important for future machine learning models to use more diverse datasets, based upon longitudinal data to evaluate whether machine learning models can identify specific features that differentiate true neurodegenerative disease from other diseases with neuroretinal implications.

## Methods

We conducted a systematic literature review utilizing two searching tools to identify datasets; Google Scholar and Ovid MEDLINE. Our search criteria included studies between January 1, 2012, and February 15, 2023, that contain image datasets with identifiable neurodegenerative disease and/ or studies that utilized deep learning analysis models.

**Figure 1.**
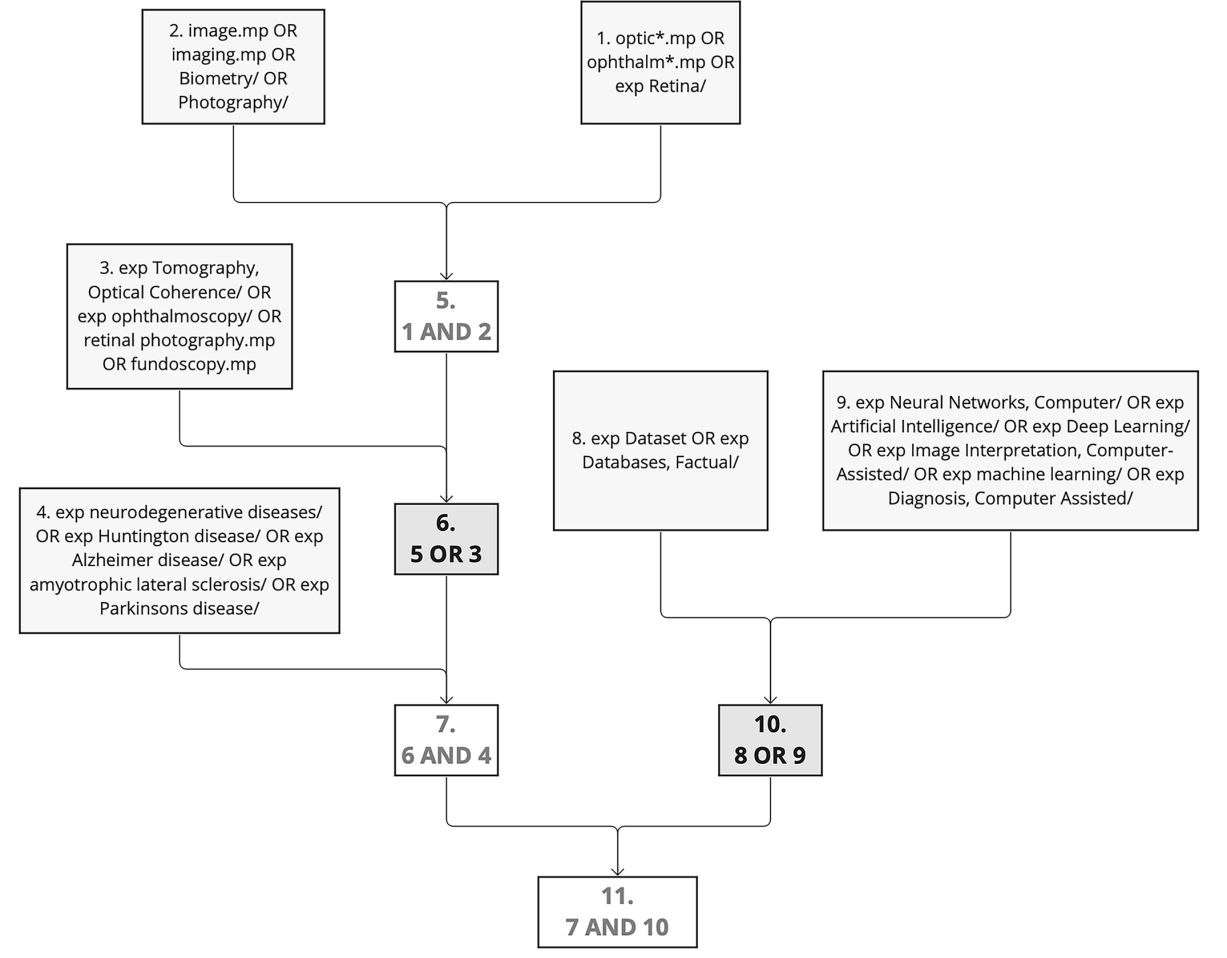
Conceptual search design as structured within Ovid MEDLINE search tool. exp, exploded, represents that the search was expanded to include similar related MeSH terms; .mp, multipurpose, indicates that all areas of the publication (title, abstract, body, etc.) were searched.

Ovid MEDLINE was used for refined searches, utilizing multi-level Boolean operators (and, or) and specific terminology (exp – explode, .mp – multi-purpose) as described below. As the final compilation of search parameters, step 11 represents final search protocol. The conceptual function of these parameters was to identify all articles with ophthalmic imaging in the context of neurodegenerative disease (steps 1–7), which also reported use of a dataset, database, or image analysis algorithm (steps 8–10). The conceptual search design is displayed in Figure 1.

1. optic*.mp or ophthalm*.mp or exp Retina/
2. image.mp or imaging.mp or Biometry/ or Photography/
3. exp Tomography, Optical Coherence/ or exp ophthalmoscopy/ or retinal photography.mp or fundoscopy.mp
4. exp neurodegenerative diseases/ or exp Huntington disease/ or exp Alzheimer disease/ or exp amyotrophic lateral sclerosis/ or exp Parkinsons disease/
5. 1 and 2
6. 5 or 3
7. 6 and 4
8. exp Dataset or exp Databases, Factual/
9. exp Neural Networks, Computer/ or exp Artificial Intelligence/ or exp Deep Learning/ or exp Image Interpretation, Computer-Assisted/ or exp deep learning/ or exp Diagnosis, Computer Assisted/
10. 8 or 9
11. 7 and 10.

Google search engine and Google Scholar were used for broad searches. The searched key terms were as follows: “retinal photography,” “neurodegenerative disease OCT,” “neurodegenerative disease fundoscopy,” “Parkinson's retina,” “neurodegenerative deep learning,” and “neurodegenerative image dataset.”

Our OVID broad search resulted in 245 studies and Google resulted in 10 studies. Additionally, we referenced prior meta-analysis studies from Chrysou et al.,^17^ Zhou et al.,^18^ Jin et al.,^19^ Chan et al.,^20^ Noah et al.,^13^ Khan et al.,^21^ Nepal et al.,^12^ and Katsimpris et al.^22^ Following our inclusion criteria, each result and dataset were independently reviewed and recorded. In the setting of incomplete information within the article, datasets were assumed to be available upon request (AoR), containing two eyes per case and one image per eye.

For the review of deep learning models, articles identified in the primary search described above were further screened according to whether they involved predictive models using multivariate data obtained from imaging, predictive models using raw images as input, or predictive models using data extracted from image feature detectors. Articles outside of this scope were excluded from the review of models presented in Section 4: “Strategies used for retinal image analysis in patients with neurodegenerative disease.”

## Results

### Dataset Summary

In total, our search yielded 154 datasets containing approximately 70,481 images of 25,053 patients with neurodegenerative disease and 10,115 healthy controls. A summary of each dataset type can be found in Table 1. See Supplementary Table S1 for the comprehensive list of datasets. AD was the most represented disease (47% of datasets, 76% of patients, 66% of controls, and 74% of images), followed by PD (34% of datasets, 10% of patients, 26% of controls, and 14% of images) and MCI (20% of datasets, 4.1% of patients, 15% of controls, and 7.0% of images), whereas D-US was the least represented (2.6% of datasets, 7.2% of patients, 0.7% of controls, and 5.6% of images). No fundus image datasets were found for PD, ALS, and HD. Two datasets were accessible within the article, one was open-access, one required an account, one was unfinished, and the rest (*n* = 149) were classified as AoR. Most datasets utilized Heidelberg (54/154) and Zeiss (56/154) devices, whereas nine other manufacturers were also represented. The majority of datasets were from publications authored in Europe (66/154), whereas Asia (35/154), the Middle East (25/154), and North America (24/154) were also well represented, and a few datasets were generated in Oceania (2/154) and South America (3/154).

**Table 1.** Summary of Retinal Image Datasets in Patients With Neurodegenerative Disease

Dataset Feature	All Datasets	AD	PD	ALS	HD	MCI	D-US
**Access type**							
Total datasets (*n*)	154	72 (47%)	53 (34%)	14 (9.1%)	8 (5.2%)	31 (20%)	4 (2.6%)
AoR^*^ (*n*/total)	149/154	69/72	52/53	13/14	8/8	30/31	4/4
Open access (*n*/total)	1/154	1/72	0/53	0/14	0/8	1/31	0/4
In article (*n*/total)	2/154	1/72	0/53	1/14	0/8	0/31	0/4
Account required (*n*/total)	1/154	0/72	0/53	0/14	0/8	0/31	0/4
**Imaging totals**							
Patients (*n*, % total)	25,053	19,035 (76%)	2563 (10%)	481 (1.9%)	141 (0.6%)	1039 (4.1%)	1794 (7.2%)
Patient eyes^†^ (n)	49,475	33,794	4858	954	280	1952	3588
Controls (*n* % total)	10,115	6717 (66%)	2636 (26%)	425 (4.2%)	155 (1.5%)	1520^§^ (15%)	72^§^ (0.7%)
Control eyes^†^ (*n*)	19,661	13,141	4994	850	310	2959	144
Images^‡^ (*n*, % total)	70,481	51,902 (74%)	9852 (14%)	2050 (2.9%)	590 (0.8%)	4911 (7.0%)	3588 (5.6%)
**Modality**							
OCT (*n*/total)	148/154	69/72	53/53	14/14	8/8	30/31	2/4
OCT-A (*n*/total)	28/154	15/72	10/53	1/14	2/8	7/31	0/4
Fundus (*n*/total)	11/154	8/72	0/53	0/14	0/8	2/31	3/4
**Device manufacturer**							
Zeiss (*n*/total)	56/154	27/72	24/53	3/14	0/8	17/31	1/4
Heidelberg (*n*/total)	54/154	19/72	18/53	9/14	7/8	5/31	0/4
Topcon (*n*/total)	10/154	5/72	2/53	2/14	0/8	1/31	0/4
Optovue (*n*/total)	26/154	15/72	7/53	1/14	2/8	5/31	0/4
Canon (*n*/total)	3/154	2/72	0/53	0/14	0/8	0/31	1/4
Other^||^ (*n*/total)	10/154	3/72	3/53	0/14	1/8	4/31	2/4
**Region** **^#^**							
North America (*n*/total)	24/154	15/72	5/53	3/14	0/8	8/31	1/4
South America (*n*/total)	3/154	1/72	1/53	0/14	1/8	0/31	0/4
Europe (*n*/total)	66/154	30/72	24/53	7/14	4/8	5/31	0/4
Asia (*n*/total)	35/154	17/72	11/53	2/14	0/8	14/31	3/4
Middle East (*n*/total)	25/154	9/72	12/53	2/14	2/8	3/31	0/4
Oceania (*n*/total)	2/154	1/72	0/53	0/14	1/8	0/31	0/4

AD, Alzheimer's disease; ALS, amyotrophic lateral sclerosis; AoR, available upon request; D-US, dementia, un-specified; HD, Huntington's disease; MCI, mild cognitive impairment; OCT, optical coherence tomography; OCT-A, optical coherence tomography angiography; PD, Parkinson's disease.

* If not specified within the article, datasets were assumed to be available upon request (AoR) from the corresponding author.

† Extrapolated two eyes per case if not specified.

‡ Extrapolated one image per eye if not specified.

§ In multiple HD and D-US case-control studies, the same controls were used for comparison against other disease types (AD, PD, and HD) as well. Therefore, these controls are included in each disease type but are not double counted in the total number of healthy controls.

|| Other: Custom system, Nidek, Optos, Opthalmika, SVision, Optopol, Opko.

# North America: United States of America and Canada; South America: Brazil, Argentina; Europe: United Kingdom, Italy, Germany, Spain, The Netherlands, Portugal, Belgium, Poland, Czech Republic, Greece, and Switzerland; Asia: China, India, South Korea, Singapore, Taiwan, and Hong Kong; Middle East: Iran, Turkey, and Israel; Oceania: Australia and New Zealand.

### Retinal Findings Common Among One or More Neurodegenerative Diseases

OCT studies have revealed similarities and differences in the retinas of patients with various neurodegenerative diseases. Table 2 contains a summary of the retinal findings organized by disease. Due to variation in results between individual studies, meta-analyses were referenced when available. In patients with AD, PD, MCI, and D-US, thinning of the pRNFL has been seen in all four quadrants, whereas the temporal and superior quadrants have been the only affected quadrants in patients with HD. Reduced macular volume, mGCIPL loss have been demonstrated in patients with AD, PD, and MCI, but not ALS or HD. Macular thinning has been identified in patients with HD, AD, PD, and MCI, but not ALS or D-US. Although the inner nuclear layer appears to be spared in patients with PD, it is reduced in patients with ALS. Choroidal thinning has been found in patients with AD, HD, and MCI, but not PD. As discussed later, these and other retinal biomarkers have been shown to correlate with disease severity and duration.

**Table 2.** Summary of Retinal Findings in Neurodegenerative Disease

Imaging Feature	Change	Studies (n)	Significance	Ref
**Alzheimer's disease**				
**Fluorescence studies**				
Beta-amyloid (curcumin)	Increased	2	2/2 with *P* < 0.05^†^	23, 24
Inclusion bodies in IPL (blue-AF)	Increased	1	*P* = 0.03	25
FLIO	Increased	1	*P* < 0.05	26
**Fundus**				
Fractal dimension				
Arterial	Decreased	4	*P* < 0.049^*^	27
Venular	Decreased	4	*P* < 0.025^*^	27
Vessel morphology				
Arterial tortuosity	Increased/decreased	2	*P* < 0.001/*P* = 0.027	28, 29
Venular tortuosity	Increased/NS	2	*P* < 0.001/*P* = 0.952	28, 29
Venular caliber	Narrowed	1	*P* < 0.001	28
**OCT**				
pRNFL thickness	Decreased	24	*P* < 0.001^*^	20
Retinal layer thickness				
mGCIPL	Decreased	6	*P* = 0.01^*^	20
mGCC	Decreased	4	*P* < 0.00001^*^	20
Inner sector of macula	Decreased	10	*P* < 0.001^*^	20
Outer sector of macula	Decreased	10	*P* < 0.001^*^	20
Macular volume	Decreased	7	*P* < 0.05^*^	20
Choroidal thickness	Decreased	5	*P* < 0.001^*^	20
**OCT-A**				
Microvascular density				
Macular whole en face superficial	Decreased	9	*P* < 0.0001^*^	19
Macular whole en face deep	Decreased	9	*P* = 0.0001^*^	19
Parafoveal superficial	Decreased	6	*P* = 0.001^*^	19
Parafoveal deep	Decreased	3	*P* < 0.0001^*^	19
Foveal avascular zone (FAZ)	Enlarged	5	*P* = 0.07^*^	19
**Parkinson's disease**				
**OCT**				
pRNFL thickness	Decreased	33	d = -0.42^*^	17
Retinal layer thickness				
mGCIPL	Decreased	11	d = -0.40^*^	17
Inner nuclear layer	NS	5	d = -0.01^*^	17
Outer plexiform layer	NS	5	d = 0.11^*^	17
Fovea thickness	Decreased	14	*P* = 0.000^*^	18
Inner sector of macula^‡^	Decreased	8	*P* < 0.05^*^	18
Outer sector of macula^‡^	Decreased	8	*P* < 0.05^*^	18
Macular volume	Decreased	9	*P* < 0.05^*^	18
Choroidal thickness	Increased/decreased	2	*P* < 0.05, *P* < 0.002	33-34
**OCT-A**				
Microvascular density				
Superficial vascular plexus (SVP)	Decreased	7	*P* = 0.002^*^	22
Foveal SVP	Decreased	5	*P* = 0.62^*^	22
Parafoveal SVP	Decreased	5	*P* = 0.12^*^	22
Foveal avascular zone	Decreased	5	*P* = 0.39^*^	22
Branching complexity				
Superficial capillary plexus	Decreased	1	*P* < 0.01	33
Deep capillary plexus	Decreased	1	*P* < 0.05	33
Retinal flow density				
Superficial	Decreased	1	*P* < 0.05	35
Deep	Decreased	1	*P* < 0.05	35

**Table 2.** Continued

Imaging Feature	Change	Studies (n)	Significance	Ref
**Amyotrophic lateral sclerosis**				
**OCT**				
pRNFL thickness	NS/decreased	11	*P* = 0.14^*^6/11 with *P* < 0.05^†^	12
Retinal layers				
mGCIPL	NS	9	*P* = 0.67^*^	12
Outer plexiform layer	NS	6	*P* = 0.79^*^	12
Inner nuclear layer	Decreased	5	*P* = 0.00^*^	12
Outer nuclear layer	NS	5	*P* = 0.16^*^	12
Macular thickness	NS	5	*P* = 0.58^*^	12
Whole retinal thickness	NS	3	*P* = 0.11^*^	12
**OCT-A**				
Retinal microvascular density	NS	1	*P* > 0.05	37
**Huntington's disease**				
**OCT**				
pRNFL thickness				
Temporal region	Decreased/NS	8	4/8 with *P* < 0.005^†^	41-48
Superior region	Decreased/NS	8	2/8 with *P* < 0.05^†^	
Macular retinal thickness	Decreased/NS	8	3/8 with *P* < 0.05^†^	41-48
Macular choroidal thickness	Decreased	2	*P* < 0.05^†^	45-46
**OCT-A**				
Retinal microvascular density	NS	2	*P* > 0.05^†^	41,46
**Mild cognitive impairment**				
**OCT**				
pRNFL thickness	Decreased	17	*P* = 0.002^*^	13
Retinal layers				
mGCIPL	Decreased/NS	9	6/9 with *P* < 0.05^†^	13
mGCC	Decreased	1	*P* < 0.05	13
Macular thickness	NS/decreased	4	1/4 with *P* < 0.05^†^	13
Foveal thickness	NS	3	3/3 with *P* > 0.05^†^	13
Choroidal thickness	Decreased	1	*P* < 0.05	44
Macular volume	NS/decreased	3	1/3 with *P* < 0.05^†^	13
**OCT-A**				
Microvascular density				
DVP, superior-nasal region	Decreased	1	*P* < 0.05	51
Superficial capillary plexus	NS	1	*P* > 0.05	52
Foveal avascular zone	NS	1	*P* > 0.05	52
**Unspecified dementia**				
**OCT**				
pRNFL thickness	Decreased/NS	2	1/2 with *P* < 0.001^†^	14, 53
mGCIPL thickness	Decreased/NS	2	1/2 with *P* < 0.003^†^	14, 53
Macular volume	NS	1	*P* > 0.05	53

AD, Alzheimer's disease; AF, autofluorescence; ALS, amyotrophic lateral sclerosis; D-US, dementia, unspecified; DVP, deep vascular plexus; FLIO, fluorescence lifetime imaging ophthalmoscopy; HD, Huntington's disease; mGCC, macular ganglion cell complex; mGCIPL, macular ganglion cell layer and inner plexiform layer; NS, not significant, NS, no statistical difference between cases and controls; pRNFL, peripapillary retinal nerve fiber layer; PD, Parkinson's disease; MCI, mild cognitive impairment; SVP, superficial venous plexus.

Databases containing fundus images of patients with MCI and D-US did not publish analysis of imaging features.

* Indicates a significance value provided by the referenced meta-analysis.

† Indicates that the *P* value of the corresponding number of studies fell within the indicated range.

‡ Inner and outer macular sectors classified according to Early Treatment Diabetic Retinopathy Study (ETDRS) guidelines.

OCT-A reveals additional information regarding the retinal vasculature of patients with neurodegenerative disease. Decreased microvascular density and an enlarged foveal avascular zone have been demonstrated in patients with AD and PD. Reduced branching complexity has also been associated with AD. Preliminary OCT-A case-control studies of patients with ALS, HD, or MCI have not found conclusive significant retinal findings. Fundus imaging case-control studies have been limited to only AD.

### Alzheimer's Disease

OCT studies have revealed significant retinal neurodegeneration in patients with AD. A 2018 meta-analysis^20^ found thinning in the pRNFL, mGCIPL, ganglion cell complex, and choroidal layers, as well as reduced overall macular volume and macular thinning in the inner and outer sectors. The mGCIPL thinning has also been shown to correlate with disease severity.^14^

Various fluorescent fundus imaging modalities have been used to visualize and quantify retinal AD pathology. Intravenous administration of curcumin, a beta-amyloid-binding fluorophore, revealed that the retinal beta-amyloid burden is doubled in patients with AD. Retinal beta-amyloid levels were linked to cortical beta-amyloid burden and reduced hippocampal volume.^23^^,^^24^ Alternatively, blue autofluorescence has been used to quantify the surface area of retinal inclusion bodies which correlates with preclinical cortical beta-amyloid burden.^25^ Finally, analysis of fluorescence lifetime imaging ophthalmoscopy revealed differences in patients with phakic AD compared to matched controls.^26^

Changes in retinal vasculature have been identified in fundus images of patients with AD. Fractal dimension (FD), a quantitative representation of vascular branching complexity, can be determined by commercially available software or expert analysis. A systematic review in 2019 found that vascular FD was decreased in four case-control fundus imaging studies of AD.^27^ In addition to reduced FD, one study found increased vessel tortuosity and narrowed venular caliber in patients with AD, although a separate study yielded contradictory findings.^28^^,^^29^

Advancements in OCT-A have revealed further information regarding vascular changes in patients with AD. A 2021 meta-analysis demonstrated an enlarged foveal avascular zone and reduced macular whole enface superficial and deep vessel densities in patients with AD.^19^ Notably, features of FD, vessel caliber, and vessel tortuosity that have been found on fundus imaging can also be evaluated using OCT-A, although systematic differences between modalities have been noted.^30^

### Parkinson's Disease

In addition to reduced dopamine levels, the retina of patients with PD also exhibits neurodegeneration. Meta-analyses of OCT studies in 2019^17^ and 2020^18^ revealed reduced pRNFL, mGCIPL, and macular thickness, as well as decreased macular volume in patients with PD. In addition, disease severity and duration have been shown to be linked with pRNFL thinning and decreased foveal thickness.^31^^,^^32^ Contradictory findings of both increased and decreased choroidal thickness have been reported, likely due to differences in image analysis.^33^^,^^34^ Fundus imaging studies have revealed that patients with retinal thinning compared to age-matched controls have an increased risk of developing PD.^35^

PD has also demonstrated an impact on retinal vasculature. A 2023 meta-analysis^22^ found that patients with PD had reduced microvascular density in the whole superficial vascular plexus (SVP), foveal SVP, parafoveal SVP, and foveal avascular zone (FAZ), and reduced branching complexity has also been implicated.^36^ Microvascular density, FD, and retinal flow density have each been used to successfully differentiate patients with and without PD.^37^^–^^39^

### Amyotrophic Lateral Sclerosis

Studies on ALS have produced conflicting results regarding retinal neurodegeneration. A 2022 meta-analysis^12^ found inner nuclear layer thickness to be the only statistically significant finding in patients with ALS. However, 6 of the 11 studies within the analysis also demonstrated significant pRNFL thinning. Future studies with differentiation between ALS subtypes (bulbar-onset versus spinal-onset) may help clarify the conflicting results.^12^ In the only found OCT-A study assessing patients with ALS, retinal microvessel density was not significantly decreased in patients with ALS compared to controls.^40^

### Huntington's Disease

OCT case-control studies of patients with HD have produced conflicting results. Temporal pRNFL deterioration and reduced macular retinal and choroidal thickness have been demonstrated in patients with HD, but other studies found no significant difference.^41^^–^^48^ One study found that temporal pRNFL thinning appeared in preclinical HD, whereas another could not replicate the finding.^44^^,^^48^ Disease duration and severity may be correlated with temporal pRNFL thinning and reduced macular volume.^42^ Two studies using OCT-A found no difference in vessel density.^41^^,^^46^ A total of 115 patients with HD have been studied, suggesting that larger sample sizes may assist in ascertaining the retinal characteristics of HD.

### Unspecified Dementia and Mild Cognitive Impairment

Patients with MCI have demonstrated retinal changes. A 2020 meta-analysis^13^ discovered pRNFL thinning across 17 studies. OCT case-control comparisons have also demonstrated decreased mGCIPL, ganglion cell complex, macular, foveal, and choroidal thickness, and reduced macular volume, although all studies did not share the same findings. GCIPL thickness,^49^ choroidal thickness,^50^ pRNFL thickness,^51^^–^^53^ and the pRNFL granular membrane area^54^ have been shown to be inversely correlated with cognitive performance, whereas other studies show no correlation.^55^ Decreased mGCIPL thickness has also been correlated with a reduction of white matter in the fornix of patients with MCI.^56^ An OCT-A study discovered reduced microvascular density in the superior-nasal region of patients with MCI, but another study did not find any differences.^57^^,^^58^

Few studies have investigated the relationship between the retina and unspecified dementia. Ferrari et al.^14^ found pRNFL thinning and GCIPL loss, with GCIPL loss being connected with AD severity. On the other hand, Pillai et al.^59^ found no statistical significance in RNFL thickness, GCIPL thickness, or macular volume between patients with unspecified dementia and healthy controls. Further studies will help explore the impact of unspecified dementia on the retina.

## Strategies Used for Retinal Image Analysis in Patients With Neurodegenerative Disease

As discussed above, recent studies have elucidated various features found in retinal imaging that are associated with neurodegenerative diseases. On the other hand, several studies have used machine learning models to detect or otherwise learn more about these diseases using retinal images alone, without any a priori knowledge. Table 3 provides an overview of studies using deep learning algorithms and other predictive models for retinal image analysis in neurodegenerative disease, whereas Supplementary Table S2 contains additional details for each model. Deep learning models can detect features from unstructured data to make predictions using that data, with no guidance apart from data examples. The most prevalent deep learning algorithm for image analysis is known as the convolution neural network (CNN). In retinal images of patients with neurodegenerative disease, some of the features learned by CNN models might be features already described in the scientific literature. Some may be observable features that have not yet been described, and others may be features that are even too subtle for a human observer to detect. CNNs have shown great promise for automating decision-making tasks using retinal images in patients with neurodegenerative disease.

**Table 3.** Deep Learning Algorithms and Other Predictive Models for Retinal Image Analysis in Neurodegenerative Disease

Disease	Model Task	Model Metrics	Dataset Amount	Image Modality	General Architecture	Limitations	Ref
AD	Classification: clinical AD or controls	Binary Accuracy 82.44%	244 patients	Color fundus	Multi-modal pipeline with each step trained separately: image quality selector, UNet for vessel segmentation, SVM classifier	• Small sample size. • Dataset known to have healthier population compared with the general population. • Substantial amount of AD may have been undocumented, including some of the controls.	^82^
AD	Classification: clinical AD or controls	• Using all inputs: AUC 0.836 (CI = 0.729, 0.943). • Using GC-IPL maps, quantitative OCT data, and patient data: AUC 0.841 (CI = 0.739, 0.943). • Using GCL-IPL maps only: AUC 0.809 (CI = 0.700, 0.919).	159 patients	OCT, OCTA,color fundus, autofluorescence fundus, patient metadata	• Each feature map, as well as quantitative imaging data plus patient metadata, were passed to respective FC layers, which then converged in one final output layer. • Apart from the multimodal model, separate models were also constructed separately for each input image modality.	• Use of cropped images may have deprived the model of potentially pertinent information in the peripheral retina. • Shallow model due to small dataset limits the complexity of feature detection. • AD patients were diagnosed clinically rather than imaging or ApoE4 genetic basis.	^62^
AD	Classification: FBB-PET (+) or (−) negative at baseline and after 24-month period in patients with subjective cognitive decline.	• Logistic classifier accuracy not reported. • Multivariate regression showed 8% and 6% higher probability of PET+ at baseline and 2 years, respectively, per 1 µm of increased inner nasal macular thickness.	129 patients	OCT	• 16 univariate logistic regression models to predict baseline PET status. • 16 univariate logistic regression models to predict amyloid PET positivity after 24 months.	• Relatively small sample size (especially for the subgroup with abnormal Aβ). • Short follow-up period relative to AD time course.	^83^
AD	Classification: clinical AD or controls.Classification: FBB-PET (+) or (−).	• AUC for clinical AD: 0.91 (95% CI = 0.81–1.0). • AUC for FBB-PET (+) 0.86 (95% CI = 0.7–1.0).	12,949 images	Color fundus and metadata	Feature fusion via concatenation of extracted features from the four images followed by an FC layer, followed by fusion of demographic features by deep bilinear transformation, followed by FC output layer.	• Still relatively small dataset (<1000 AD patients). • Labeling based on clinical diagnosis. • Substantial overlap between Alzheimer's disease and cerebrovascular disease (shared risk factors).	^61^
AD	Classification: clinical AD or controls.	• 99% sensitivity and specificity. • Per-pixel 99.65% binary accuracy for OCT segmentation.	100 images	OCT RNFL	Radial basis function-based neural network classifier	• Small dataset. • Wavelet networks are not well-described in the literature. • Stage of AD and diagnostic criteria unclear.	^84^
AD	Classification: AD or controls	AUC of 0.79 using the I2 region of interest	39 patients	OCT RNFL and snapshot hyperspectral retinal imaging	8 different linear discriminant classifiers trained independently using normalized hyperspectral data from 4 fundus regions of interest and either fused or not fused with OCT RNFL thickness measurement data.	• Lack of biomarker confirmation of AD in 10/17 subjects. • Snapshot imaging obtaining spatial and spectral imaging at once sacrifices the resolution of both modalities.	^85^
AD	Classification: AD or controls	AUC of 0.965 for validation set	Training: 212 patients,Validation: 100 eyes	OCT RNFL	Linear discriminant classifier	• Inclusion criteria of advanced AD was used to enhance the likelihood of OCT measurements being diagnostically useful, but limits the generalizability of the model.	^86^
MCI	Classification: MCI, D-US, or controls	• AUC for MCI prediction on validation set: 0.87. • AUC for D-US prediction on validation set: 0.86.	332 images	Color fundus	• Histogram of oriented gradient was used for feature extraction. • SVM classifier.	• Single-center data lacking external validation. • Cross-sectional rather than longitudinal data. • Treats different subtypes of the stroke equally although various types may lead to different degrees and domains of cognitive impairment.	^87^
MCI, AD	Segmentation: mapping retinal vessels in OCTA images	• Per-pixel AUC of 0.95 for superficial vascular complex segmentation. • Per-pixel AUC of 0.97 for deep vascular complex segmentation.	117 images	OCTA image	A coarse stage followed by a fine stage. Throughout both stages, pixel-level and centerline-level vessel segmentation branches run in parallel. The output layer fuses the pixel-level and centerline-level branches.	• AD diagnosis was based on the NINCDS-ADRDA criteria, not amyloid PET or CSF studies. • OCTA is known to have projection artifacts.	^88^
PD	Regression: retinal age gap (predicted - actual age) and demographic covariates for 5-year incident PD risk prediction	AUC 0.708 for 5-year incident PD risk prediction	35,917 patients	Color fundus photos and patient metadata	Cox proportional hazards multivariate regression model using predicted retinal age gap and other patient covariates	• UK Biobank under-represents older, more ill members of the population. • Limited number of incident PD cases. • Unable to investigate dynamic changes in retinal age gap and incident PD.	^35^
PD, AD	Classification: AD, PD, or control	Median sensitivities of 79.5%, 77.8%, and 88.7% for AD, PD, and controls respectively.	75 patients	OCT	• SVM classifier. • 18 independent binary SVM models were developed, one per each of the six retinal layers at study and between any two of the three possible groups at study: the control, AD, and PD groups.	• Unclear whether there are retinal layer thickness differences between controls and AD or PD patients; the data features may not provide much signal. • Retinal thickness differences are more likely to be present at advanced stages of disease.	^75^
CAIDE risk score	Regression: estimate the CAIDE risk scoreClassification: high dementia risk (CAIDE > 10).	• R = 0.76 for predicted versus actual CAIDE score in the external validation set • AUC 0.926 (CI = 0.913 – 0.939) on external validation dataset for identifying individuals with high dementia risk.	• Training: 271,864 patients • Validation: 20,690 patients	Color fundus	InceptionResNetV2	• Educational level and physical inactivity were interpolated from other data (sex, age, BMI). • Significant age difference between the training and validation datasets. • Only included Chinese participants, limiting generalizability to other ethnicities.	^66^
Cognitive Scores	Regression: predict cognitive scores in healthy volunteersClassification: APOE4 gene (+) or (−)	• Fundus images and metadata only explained 22.4% of the variance in cognitive scores in this model. • AUC 0.47 for APOE4 classification	• 25,737 images labeled with cognitive scores • 26,622 images labeled with APOE4 genotype	Color fundus and patient metadata	Pretrained network (Inception V3, MobileNet V2 or EfficientNetB3) extracted image features, concatenated with patient metadata, then passed through two FC layers, the last of which being the output layer.	• The study population under-represented individuals with severe cognitive impairment (only healthy individuals were included) • Study was limited to the Canadian population • Metadata was self-reported, potentially introducing bias	^65^
Age	Regression: predictage in healthy aging individuals	R = 0.81 comparing predicted and actual age	19,200 images	Color fundus	Xception convolutional neural network- a 71-layer-deep pretrained network using ImageNet database similar to inception V3 but with depthwise-separable convolutions.	• Cross-sectional nature of the study rather than longitudinal does not allow for investigation of changes in trajectory • UK biobank comprised of more healthy population compared with general population	^68^
White matter hyperintensities	Detect white matter hyperintensities with age-related white matter change (ARWMC) score > 2 and classification of brain region to one of 6 potential regions	AUC: 0.955 based on 10-fold cross-validation for detection of ARWMC > 1 across any of the 6 brain regions	240 patients	Color fundus	* Parallel convolutional neural network models ResNet50 (for pixel-based feature extraction) and ARIA (for texture, spectral, and fractal feature extraction). * Extracted features passed to Glmnet, then SVM classifier	• Small sample size • Regional white matter hyperintensity load in the study group is not as high as in other patient groups.	^64^

AD, Alzheimer's disease; ALS, amyotrophic lateral sclerosis; ARIA, automatic retinal image analysis; ARWMC, age-related white matter change score; CAIDE, Cardiovascular Risk Factors, Aging, and Incidence of Dementia; D-US, dementia unspecified; HD, Huntington's disease; MCI, mild cognitive impairment; mGCIPL, macular ganglion cell layer and inner plexiform layer; 95% CI, 95% confidence interval; PD, Parkinson's disease; pRNFL, peripapillary retinal nerve fiber layer; SVM, support vector machine; SVP, superficial venous plexus.

An excellent review recently detailed the computational strategies for using CNNs for retinal image analysis in general, which are applicable to the current discussion. The authors outlined the following stepwise elements in the overall framework for implementing a deep learning model for retinal image analysis. These include: (1) image acquisition and annotation, (2) retinal image preprocessing, (3) model architecture and design, (4) model training, (5) generating model predictions, and (6) evaluating model performance.^60^ For a more detailed explanation of these stages, we refer the reader to this other review.^60^ In the current work, we will discuss specific architectural elements as they pertain to the problem of retinal image analysis for neurodegenerative disease, and their past and future applications.

### CNN Models: Basic Architectures and Applications

**Figure 2.**
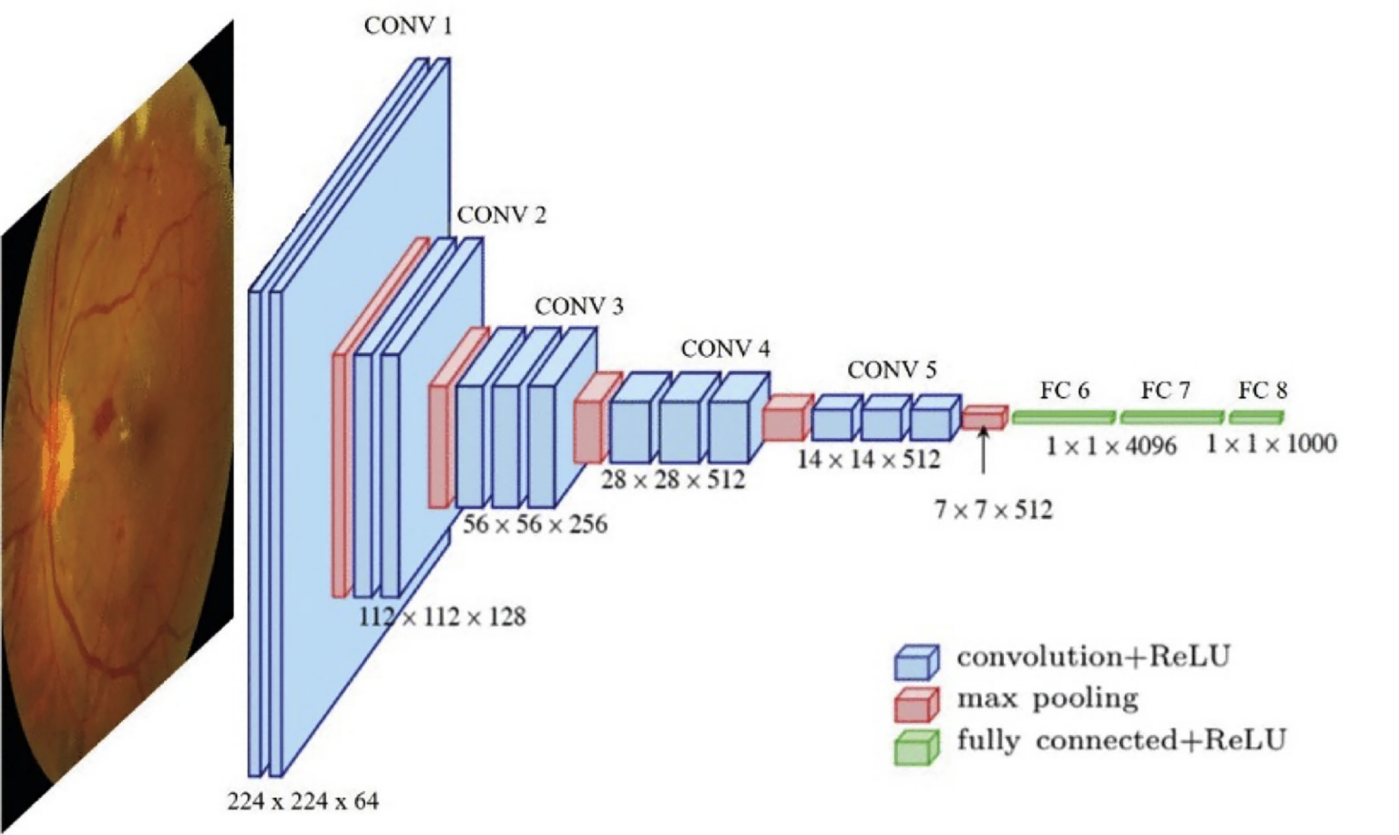
Architecture of VGGNet. Used with permission from Goutam et al. 2022.^60^ CONV, a conversion of the convolutional neural network; FC, fully connected layer; ReLU, rectified linear unit.

The architecture of a neural network refers to the arrangement of computational steps, which are known as layers. CNNs share a basic unit, the convolution layer, which is used to extract information from images. Computations in convolutional layers are fed forward in series to other types of computational layers including pooling and fully connected (FC) layers, and also other downstream convolution layers. The most common types of computational tasks performed by CNNs are classification, regression, and segmentation. The architecture of VGGNet, a common CNN used for retinal image analysis, is shown in Figure 2.

#### Classification

The structure of a basic CNN for the task of image classification usually involves several repeated units which consist of one or more convolution layers followed by a pooling layer. With each iteration of units, the resolution of the output decreases. Feed-forward skip connections are used throughout to preserve important spatial data from earlier steps. Finally, an FC layer is used to transform the spatial image data into a structured classification output. Classification models have been the most common type of CNN among publications on retinal image analysis in the context of neurodegeneration. Accuracy and area under the receiver operator curve (AUC) are the most popular methods for measuring the performance of these models, but binary accuracy, sensitivity, and specificity are also used.

Disease detection is the most basic classification task; many different models have been constructed to take images of a fundus as input and provide as output a binary label distinguishing whether the image is from a patient with a neurodegenerative disease or from a healthy control. Attempts at detecting AD, PD, and general cognitive impairment using fundus photographs have been described previously, with varied results. CNN models trained on large fundus datasets have demonstrated improved performance compared to models trained on small datasets. For example, a model trained on nearly 13,000 images had an AUC of 0.91,^61^ whereas a model trained on less than 300 images had an AUC of 0.83.^62^ Ongoing work is being done to engineer these models to improve their diagnostic accuracy. Multi-class disease detection models have also been constructed. These aim to detect one of several potential disease states; for example, one study used support vector machines (a different type of machine learning model) to classify patients as having AD, PD, or neither.^63^ Classification is not limited to the diagnosis of disease and may involve multiple classes representing any variable of interest. For example, one study devised a method for classifying the localization of age-related white matter changes to one of six overall brain regions using bilateral fundus photos fed to a CNN, followed by a regression and decision tree for brain region classification.^64^ This approach was able to classify the location of white matter lesions into one of 6 potential regions: left and right frontal lobes, parietal–occipital lobes, or basal ganglia using fundus photographs, with an AUC of 0.955 based on 10-fold cross-validation.

Risk stratification is another classification task which involves labeling imaging as belonging to one or two groups of varying risk. Many such studies have attempted to predict the presence of a known risk marker. For example, one model was trained to detect the ApoE4 genotype using fundus photographs. However, this model was unsuccessful with an AUC of 0.47, which may have been due to the low representation of ApoE4 individuals in the dataset, the model structure itself (although the model had good performance on age and sex predictions), or alternatively the inability of fundus photography as a modality to capture information about retinal amyloid deposits.^65^

#### Regression

A very similar type of CNN model can perform a regression task using image inputs. Regression models have the same general framework as classification models, except for the final computational layer. In a regression model, the final layer outputs a numeric variable (rather than categorical variables used in classification). The correlation value R is most commonly used to describe what percent of the variability in the dependent variable can be explained by the model.

For instance, one model attempted to predict cognitive scores in a cohort of aging adults, although the model was only able to explain 22% of cognitive scores (R = 0.22).^65^ Another study with a similar objective of predicting the Cardiovascular Risk Factors, Aging, and Incidence of Dementia (CAIDE) yielded a more useful model, with R = 0.76 on an external validation set and an AUC of 0.926 for the detection of high dementia risk defined as a score >10.^66^ One of the more striking successful examples of regression using CNNs is the estimation of a person's age using a fundus photograph, a biomarker that is known as retinal age, with R = 0.81 in one study.^67^^,^^68^ Other potential applications of regression are hazard and time-risk models. For instance, one model used the retinal age gap (the difference between retinal age and true age) and other demographic data as input into a Cox proportional hazards model to estimate the 5-year incidence of PD.^35^

#### Segmentation and Object Detection

Image segmentation is a different kind of task that uses a very different model architecture. The purpose of a segmentation algorithm is to use an input image to create a segmentation map, which is an image highlighting every pixel from the input image that is associated with a structure of interest. Similar to other CNN models, segmentation models use blocks of convolutional and pooling layers. In a segmentation model, intermediate convolution layers may scale down the resolution of the image data, but up-convolution layers are used to increase the output resolution (back to the original resolution in many cases). Feed-forward skip connections are used to preserve important spatial data from the higher-resolution steps to create the segmentation map.

Segmentation of the retinal vessels is one of the most important examples of this type of algorithm, as the vascular anatomy contains valuable information regarding the neurodegenerative disease, as noted above. However, segmentation of retinal vessels has also been described as one of the most challenging tasks in retinal image processing.^69^ Several segmentation algorithms have been developed over the past few years with the objective of generating images of the retinal vascular tree from fundus images, with all other details removed from the image. A vascular segmentation algorithm for OCT-A images has also been developed recently.^70^ In practice, these models can be used to extract from the original image an image of white vessels only on a black background (or vice-versa). Many studies have used pre-existing vessel segmentation algorithms to process input images and use the resulting vessel map alongside the original image as a second input to a classification or regression model. A standardized method has recently been described for evaluating and comparing the accuracy of vessel maps. Using this method, a per-pixel (vessel or no vessel present in this pixel) AUC can be calculated by averaging over every pixel in an entire image set.^70^

Object detection shares some similarities with segmentation but also has some elements of a classification model. These algorithms can detect and quantify one or more features in a fundus photograph. For example, the automated retinal image analysis (ARIA) algorithm has been used to detect and count arteriole-venous nicking, arteriole occlusions, hemorrhages, and exudates,^64^ and these results can be used as input features in a model.

### Architectural Modifications and Other Tools for Increasing Model Performance

Presently, most studies using retinal images of patients with neurodegenerative disease have been relatively small in size (in the tens to hundreds of data examples) compared with datasets more commonly used for commercial deep learning applications (often in the thousands to over a million data examples). Ample large-scale datasets exist for diseases such as diabetic retinopathy or glaucoma, but there is a scarcity of available retinal image data for patients with neurodegenerative disease. There are some moderately sized datasets for AD and several small datasets that could potentially be pooled. However, sizable retinal image datasets from patients with other common neurodegenerative diseases with lower prevalence like ALS, HD, or frontotemporal dementia are greatly lacking.

The term “Hand-engineering” refers to the use of a priori knowledge to intentionally construct a model with specific elements to increase its likelihood of recognizing features that are already known to be important in the task to be automated. Hand engineering a model by incorporating various deep learning tools into the model architecture can increase the predictive power of CNN models, especially for small datasets, although these methods can also increase the accuracy of models trained on large datasets as well.

In this section, we discuss a few of the most important deep learning tools currently available to allow for the harnessing of a priori knowledge, or otherwise increase the power of models used to analyze retinal images from patients with neurodegenerative disease.

#### Image Preprocessing and Feature Extraction

One of the simplest means of hand engineering can be preprocessing data to enhance specific features. Different combinations of image color, contrast, noise, or sharpness modifications have proven helpful for improving the visualization of darker vessels, the brighter optic nerve head, or retinal background lesions, for example.^60^ In small datasets in which images from only one eye are input at a time, it can be helpful to horizontally flip all images of left eyes to match the right eyes to increase dataset homogeneity.

Increasing the size of a dataset, known as data augmentation, can also improve model performance. For image datasets, this is commonly done using image transformations. The addition of randomly flipped, rotated, cropped, zoomed, or blurred copies of existing images in the dataset can improve model performance. Non-random cropping can also be useful, to focus on certain regions of interest, such as the optic nerve head or macula.^60^

Pre-existing deep learning algorithms can also be used to transform an image during preprocessing. Several powerful retinal vessel segmentation algorithms have been developed recently. These tools have the ability to generate segmentation maps isolating the arteries, veins, or both.^69^ Other segmentation algorithms can detect and create labeled maps of pathologic features including hemorrhages, microaneurysms, exudates, or retinal neovascularization.^71^ These algorithms were specifically developed with the small-dataset problem in mind. Even a small-scale model can obtain key lesion information reliably if the inputted images are preprocessed by a pre-existing model that can detect these lesions accurately.

Segmentation algorithms can also be used in the preprocessing of OCT images to identify the various retinal layers or create maps of their spatial thickness distribution. For example, segmentation of OCT layers can be used to generate several 2D maps of the thickness of each retinal layer, which can be used as input into a CNN. This is important feature information, given the known association between neurodegenerative disease and retinal layer thinning.

Feature extraction is the practice of using computations on original input data, which may include pre-existing neural network models, but also simpler calculations, to extract desired data features. This can be done with pretrained CNNs specialized for extracting a specific type of feature of interest (i.e. vascular information). For example, retina-based microvascular health assessment system (RMHAS) is an algorithm that can extract the following features from a retinal image: average vessel diameter, average vessel length, fractal dimension, branching angles, tortuosity, branching coefficient, asymmetry ratio, junctional exponent deviation, and angular asymmetry.^72^ Several other models are available for extracting various quantitative features from retinal images. The extracted feature data can then be used as input to a model. Occasionally, when features of interest are unknown, feature extraction can be done using the pretrained weights from early layers of a general image classifier (such as ImageNet), although the outputs from this form of feature extraction are difficult to correlate with any physiological characteristics.

One potential pitfall of both preprocessing and feature extraction is the loss of data due to the transformation of the original image data. For example, cropping to focus on the optic disk would result in a loss of data for the entire rest of the fundus. Likewise, inputting only segmented images of the vascular tree would neglect the rest of the retinal background. In addition, by the same token, inputting only structured data obtained from feature extraction would neglect any other features potentially contained in the image. For larger datasets, it is usually best to use the original images as parallel inputs together with the preprocessed images to avoid the loss of features within the original image, although for smaller datasets this may not negatively impact model performance. The methods for merging parallel inputs, known as feature fusion, will be discussed in another section below.

#### Transfer Learning

In the previous section, we discussed how the use of pre-existing models can be a powerful tool when hand-engineering a model. This is also true for training the model itself. The task of basic recognition of curves, outlines, and shapes is not trivial and must be learned by a naive model. Rather than training a naive model, the more common practice is to begin with a pretrained network, load its learned weights, and then fine-tune the weights of a few final layers during the specialized training for the specific task at hand. Importantly, the use of transfer learning was shown to yield better results compared with naive model training for predicting systemic information using retinal images, even for naive models trained on very large datasets.^73^ Examples of common pretrained CNN models include ResNet (Microsoft), VGGNet (Oxford), ImageNet (ImageNet), EfficientNet (Google), and GoogLeNet/ Inception (Google).

Using pretrained networks can be problematic, however. Pretrained CNNs tend to favor features that were important for classifying images from their original training datasets. For example, many pretrained CNNs are known to favor texture features over shape features.^74^ Although texture, or local patterns distributed over a small area, seems to be very important for neurodegenerative disease classification,^75^ it may be a pitfall to neglect key shape information, which is associated with objects across a larger area in the image. One solution to this problem is the use of pretrained networks that prioritize shape, such as Stylized-ImageNet, which used different training data to intentionally emphasize shape features over texture.

#### Feature Fusion

Feature fusion is the merging of features from different branches or different layers in a model so that the model will consider them together in one or more computational steps. Commonly, this is accomplished by simple concatenation (appending) or summation (adding). Feature fusion can be used to combine parallel branches (i.e. fuse patient metadata with a fundus image) or within elements (i.e. between convolution layers downstream of a single input image).

One important application of feature fusion to the current discussion is the simultaneous consideration of bilateral fundus images. It seems particularly important to consider data from both eyes when using fundus images to predict extraocular disease states, such as neurodegenerative disease. Several recent studies have described various strategies of feature fusion for the analysis of bilateral fundus images. Recently, a powerful model using feature fusion of bilateral fundus images achieved >99% precision and >99% recall classifying 8 different disease classes, including systemic diseases like diabetes and hypertension using retinal images from the ODIR dataset (*n* = 5000).^76^

**Figure 3.**
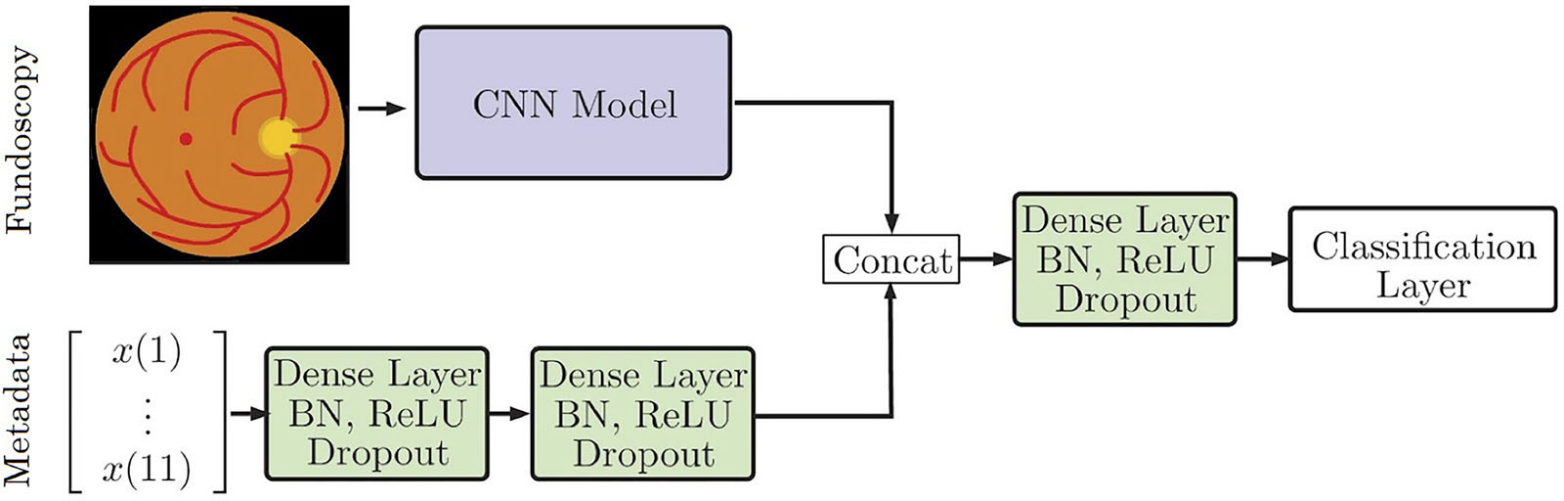
Simple approach for combining fundoscopic image processing and metadata processing. Adapted with permission from Gessert et Al. 2020.^77^ CONV, a conversion of the convolutional neural network; FC, fully connected layer; ReLU, rectified linear unit.

Another possibility with feature fusion is the parallel use of metadata which may include demographic information, like sex, age, ApoE genotype, cardiovascular laboratory markers, or potentially any information from the medical history. These data seem especially important for giving systemic contextual information and has proven useful in several models. For example, the use of metadata fused with imaging by simple concatenation improved the accuracy of skin cancer classification using dermoscopy images (see Figure 3).^77^ Similarly, the detection of anemia using fundus imaging together with metadata was significantly more accurate compared to either alone.^78^

#### Attention Mechanism for CNNs

The attention mechanism is a deep learning tool that was first developed in language models, giving them the ability to maintain attention to key parts of a sentence that were closely linked either grammatically or semantically despite being distant from one another (many words apart within a sentence or paragraph). For CNNs, the mechanism of spatial attention is the ability to give more weight to certain regions of the input image and less to others, despite being distant from one another, and the attention function is learnable by the model during training rather than hard-coded.

A new method for incorporating attention into feature fusion has been described recently.^79^ Attentional feature fusion allows a model the ability to preferentially give attention to different features rather than giving equal attention to different features, which is the result of simple methods like concatenation and summation.

#### Class Activation Mapping: Shedding Light on Influential Features

A major drawback to the use of deep neural networks for image analysis has been their black-box nature, or, in other words, their inability to explain their decision-making processes. Class activation mapping (CAM) is a method of highlighting the key features either in the original image or in any of the convolutional layers that are most influential in a model's decision and can be used for classification and segmentation tasks. This is a method of inspecting a convolution layer's implicit attention by displaying a map of the relative weights for each pixel. Self-Attention class activation maps SA-CAM^80^ have recently been described as a new method by which spatial attention maps can be visualized, which is useful for displaying explicit rather than implicit model attention.

**Figure 4.**
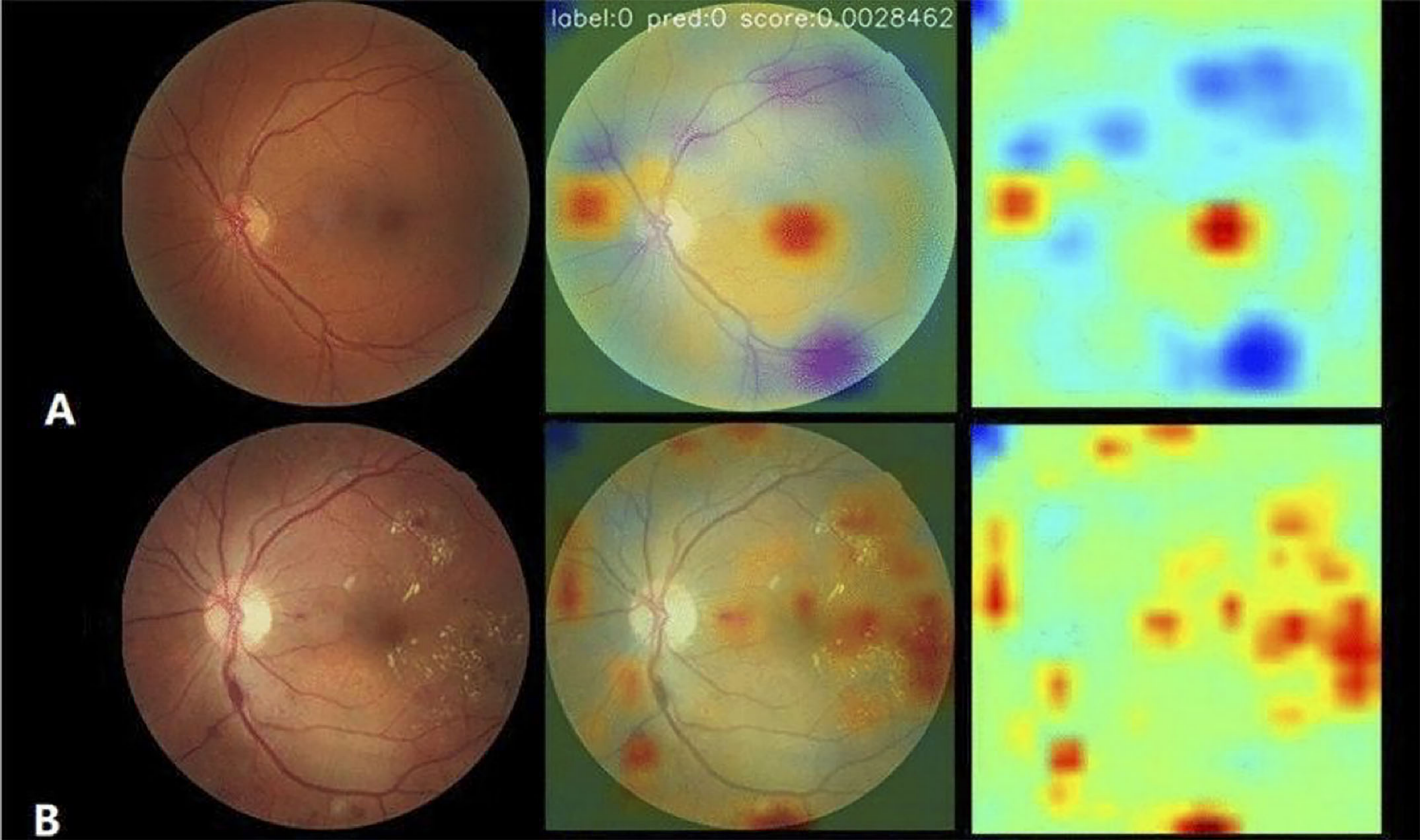
Example of attention maps used in the classification of diabetic retinopathy. Pixels in the image that are of higher relevance to model decision making are highlighted in *yellow* and *red*. Adapted with permission from Zhang et al. 2022.^81^

The benefit of using CAM is that it may help find meaningful clinical correlations of model behavior. In diabetic retinopathy models, attention maps have shown model attention to lesions known to be important for Early Treatment Diabetic Retinopathy Study (ETDRS) classification of diabetic retinopathy, such as microaneurysms and hemorrhages (Figure 4).^81^ There is currently no known classification system for neurodegenerative disease based on retinal image findings. However, CAM in a model for the detection of symptomatic AD showed that the model gave attention to areas of decreased vessel density in the fovea and temporal macula in images classified as positive for AD.^62^ Making sense of the features used by CNNs in their decision making will be important for linking model predictions with physiologic features.

## Discussion

In the recent literature, a variety of studies have demonstrated that retinal image analysis using deep learning can provide highly useful predictions regarding neurodegenerative disease diagnosis and risk assessment with good accuracy. Using retinal fundus photographs and/or OCT images, convolutional neural network algorithms have performed impressive tasks such as automated diagnosis of AD and PD, calculation of risk of future PD or dementia, and localization of cerebral white matter disease.

Recent advances in deep learning should be considered in the development of future models. For retinal image analysis in general, transfer learning, or the use of a convolutional neural network with pretrained weights, seems to be superior to training a model with naive weights, even when a large fundus image dataset is available for training. New advances, such as attentional feature fusion, will also allow for improved ability for researchers to understand model decision making. Ongoing development of feature extraction algorithms is enabling the generation of improved maps of lesions and vasculature, as well as the generation of structured feature information such as retinal age or whole-fundus vessel caliber. However, the use of extracted feature information together with original images yields better results than extracted features alone.

Because the performance and generalizability of convolutional neural network models tend to improve as more images are used during training, the predictive power of future models is expected to increase as more retinal image data becomes available in patients with neurodegenerative disease. Several existing datasets are available and contain various imaging modalities primarily in patients with AD, although dataset size for most of these is on the order of tens to a few hundred. There is very little image data available for PD, HD, ALS, or other neurodegenerative diseases. At present, although cross-sectional imaging data is becoming more abundant, datasets with longitudinal imaging are altogether lacking. Furthermore, the use of bilateral and multimodal imaging together with metadata seems to improve model performance, thus larger multimodal datasets with patient metadata and bilateral images are also needed.

Thus far, a broad range of device manufacturers have been represented in datasets. It could certainly be a pitfall to deploy a machine learning model to interpret images acquired using devices different from those used for model training. This could lead to unpredictable model behavior. However, using images from different devices and manufacturers together in the same training set would not be expected to detract from the rigor of a model as long as the images in the test dataset were acquired using one of the devices used for training. In fact, a model trained using images from several different devices or manufacturing systems could have better generalizability than a model trained on images from a single device.

Although deep learning can potentially be a powerful tool for neurodegenerative disease, virtually all models in the current literature share a similar limitation. Features such as retinal thinning are highly nonspecific and could represent a variety of pathologies, such as glaucoma, diabetes, or other inflammatory retinopathies. In addition, whereas many reports have claimed to detect neurodegenerative diseases with high specificity and sensitivity, most of these datasets are poorly representative of a realistic clinical population. It will be important for future models to use more diverse datasets that do not exclude disease with ocular manifestations and re-evaluate whether these models can identify features differentiating true neurodegenerative disease from other diseases with retinal implications. Given these limitations, it remains uncertain whether automated retinal image analysis using machine learning algorithms will be useful for the diagnosis of neurodegenerative disease in clinical practice.

## Supplementary Material

Supplement 1

Supplement 2
