# Supplementary material for: Deep Learning and Machine Learning Algorithms for Retinal Image Analysis in Neurodegenerative Disease: Systematic Review of Datasets and Models
Source: Transl Vis Sci Technol. 2024 Feb 21;13(2):16. doi: 10.1167/tvst.13.2.16 (PMC10893898; doi:10.1167/tvst.13.2.16)
Supplement: Supplement 1 [file tvst-13-2-16_s001.docx]

**Supplementary Table 1:**
A comprehensive list of datasets with retinal imaging of patients with neurodegenerative disease.

AD, Alzheimer’s disease; PD, Parkinson’s disease; ALS, amyotrophic lateral sclerosis; HD, Huntington’s disease; MCI, mild cognitive impairment; D-US, dementia, un-specified; ctrls, controls; AoR, available upon request; OCT, optical coherence tomography; OCT-A, optical coherence tomography angiography; SD, spectral domain; FD, Fourier domain; FA, fluorescein angiography; mGCIPL, macular ganglion cell layer and inner plexiform layer; pRNFL, peripapillary retinal nerve fiber layer.

| **Dataset or publication name** | **Disease** | **Access type** | **Data access details** | **Authors Country of origin** | **# of patients** | **# of eyes** | **# of images** | **Modality** | **Device** | **Reference** |
| --- | --- | --- | --- | --- | --- | --- | --- | --- | --- | --- |
| Machine learning for comprehensive prediction of high risk for Alzheimer’s | AD | AoR | Ygal.rotenstreich@sheba.health.gov.il | Israel | 125 |  |  | OCT | Zeiss | ^1^ |
| Convolutional neural network to identify symptomatic Alzheimer's disease using multimodal retinal imaging | AD | AoR | sharon.fekrat@duke.edu | USA | 159 |  |  | OCT OCTA Fundus photography and FA | Zeiss and Optos | ^2^ |
| Association between retinal thickness and β-amyloid brain accumulation in individuals with subjective cognitive decline: Fundació ACE Healthy Brain Initiative | AD | AoR | mmarquie@fundacioace.org | Spain | 129 |  |  | OCT | 3D OCT Maestro, Topcon | ^3^ |
| UK Biobank | AD | Account | www.ukbiobank.ac.uk/ | United Kingdom | 87 |  | 122 | OCT, Fundus photography | Various | ^4^ |
| Longitudinal retinal layer changes in preclinical Alzheimer’s disease | AD | AoR and Link | ja.vandekreeke@amsterdamumc.nl | Netherlands | 145 |  |  | SD-OCT | Heidelberg Spectralis | ^5^ |
| Evaluation of choroidal thickness in prodromal Alzheimer’s disease defined by amyloid PET | AD | In article | alicialeguileta@gmail.com casadorojo@hotmail.es | Spain | 34 AD 51 ctrls | 63 AD 51 ctrls |  | SD-OCT | Spectralis | ^6^ |
| Evaluation of optical coherence tomography angiographic findings in Alzheimer’s type dementia | AD | AoR | bulutme73@yahoo.com | Turkey | 26 AD  26 ctrls |  |  | OCT-A | Optovue | ^7^ |
| Retinal amyloid pathology and proof-of-concept imaging trial in Alzheimer’s disease | AD | AoR | maya.koronyo@cshs.org koronyoy@cshs.org | USA | 16 AD  6 ctrls |  |  | OCT | Heidelberg Spectralis | ^8^ |
| Canadian Longitudinal Study on Aging (CLSA) database | AD | AoR | <http://www.clsa-elcv.ca> for researchers who meeting access criteria. | Canada | 14,711 |  | 25 737 | OCT  Fundus photography | Various | ^9^ |
| Analysis of optic disk color changes in Alzheimer's disease: A potential new biomarker | AD | AoR | egmvivax@yahoo.com | Spain | 56 AD  56 ctrls |  |  | OCT | Zeiss | ^10^ |
| Evaluation of the chorioretinal thickness changes in Alzheimer's disease using spectral-domain optical coherence tomography | AD | AoR | alihasanbayhan@hotmail.com | Turkey | 31 AD  30 ctrls |  | 61 | SD-OCT | RTVue-100 | ^11^ |
| Macular Thickness Measurements with Frequency Domain-OCT for Quantification of Retinal Neural Loss and its Correlation with Cognitive Impairment in Alzheimerʼs Disease | AD | AoR | leonardo_provetti@yahoo.com.br | Brazil | 48 AD  24 ctrls |  | 93 | FD-OCT | Topcon Corp., Tokyo, Japan | ^12^ |
| OCT in Alzheimer’s disease: thinning of the RNFL and superior hemiretina  Choroidal thinning: Alzheimer's disease and aging | AD | AoR | cunha.oft@gmail.com | Portugal | 50 AD  152 ctrls |  | 202 | SD-OCT | Spectralis Heidelberg | ^13,14^ |
| Neurodegeneration in ocular and central nervous systems: optical coherence tomography study in normal-tension glaucoma and Alzheimer disease | AD | AoR | muhsineraslan@hotmail.com | Turkey | 20 AD  20 ctrls |  |  | OCT | Optovue | ^15^ |
| Ganglion cell layer measurements correlate with disease severity in patients with Alzheimer's disease | AD | AoR | egmvivax@yahoo.com | Spain | 75 AD  75 ctrls |  |  | OCT | Spectralis | ^16^ |
| Choroidal Thinning as a New Finding in Alzheimer's Disease: Evidence from Enhanced Depth Imaging Spectral Domain Optical Coherence Tomography | AD | AoR | magda.gharbiya@tiscali.it | Italy | 21 AD  21 ctrls |  |  | SD-OCT |  | ^17^ |
| Retinal vascular and structural changes are associated with amyloid burden in the elderly: ophthalmic biomarkers of preclinical Alzheimer’s disease | AD | AoR | mojtaba.golzan@uts.edu.au | Australia | 28 AD  73 ctrls |  |  | OCT | Nidek Co., Gamagori, Japan | ^18^ |
| Evaluation of the chorioretinal thickness changes in Alzheimer disease using spectral-domain optical coherence tomography | AD | AoR | dralimesefer@hotmail.com | Turkey | 40 AD  40 ctrls |  |  | SD-OCT | RTVue | ^19^ |
| Retinal Nerve Fiber Layer Thickness in Patients With Alzheimer Disease | AD | AoR | drskirbas@gmail.com | Turkey | 40 AD  40 ctrls |  |  | SD-OCT | Unspecified | ^20^ |
| Detection of Retinal Nerve Fiber Layer Defects in Alzheimer’s Disease Using SD-OCT | AD | AoR | beutelspacher@urz.uni-heidelberg.de | Germany | 22 AD  22 ctrls |  |  | SD-OCT | Spectralis Heidelberg | ^21^ |
| Potential New Diagnostic Tool for Alzheimer's Disease Using a Linear Discriminant Function for Fourier Domain Optical Coherence Tomography | AD | AoR | egmvivax@yahoo.com | Spain | 151 AD  61 ctrls |  | 212 | OCT | Zeiss  Spectralis Heidelberg | ^22^ |
| Evaluation of Retinal Nerve Fiber Layer and Ganglion Cell Layer Thickness in Alzheimer's Disease Using Spectral-Domain Optical Coherence Tomography | AD | AoR | giovanni.staurenghi@unimi.it | Italy | 21 AD  21 ctrls |  |  | SD-OCT | Optovue  Spectralis Heidelberg | ^23^ |
| Retinal nerve fiber layer thinning in dementia associated with Parkinson's disease, dementia with Lewy bodies, and Alzheimer's disease | AD | AoR | jbenitol@meditex.es | Spain | 10 AD  10 ctrls |  |  | OCT |  | ^24^ |
| Reliability and validity of Cirrus and Spectralis optical coherence tomography for detecting retinal atrophy in Alzheimer's disease | AD | AoR | egmvivax@yahoo.com | Spain | 75 AD  75 ctrls |  | 150 | OCT | Zeiss  Spectralis Heidelberg | ^25^ |
| Visual dysfunction and its correlation with retinal changes in patients with Alzheimer's disease | AD | AoR | mariasatue@gmail.com | Spain | 24 AD  24 ctrls |  | 48 | SD-OCT | Zeiss | ^26^ |
| Analysis of Retinal Peripapillary Segmentation in Early Alzheimer's Disease Patients | AD | AoR | ramirezs@med.ucm.es | Spain | 23 AD  28 ctrls |  |  | OCT | Topcon, Japan | ^27^ |
| Retinal nerve fibre layer thickness changes in Alzheimer's disease: Results from a 12-month prospective case series | AD | AoR | alessandro.trebbastoni@uniroma1.it | Italy | 36 AD  36 ctrls |  |  | OCT | Spectralis Heidelberg | ^28^ |
| Attenuation of Choroidal Thickness in Patients With Alzheimer Disease Evidence From an Italian Prospective Study | AD | AoR | alessandro.trebbastoni@uniroma1.it | Italy | 39 AD  39 ctrls |  |  | SD-OCT | Spectralis Heidelberg | ^29^ |
| Combination of snapshot hyperspectral retinal imaging and optical coherence tomography to identify Alzheimer’s disease patients | AD | AoR | sophie.1.lemmens@uzleuven.be | Belgium | 10 AD  7 amyloid-proven AD  22 ctrls |  |  | OCT | Optovue | ^30^ |
| Analysis of Retinal OCT Images for the Early Diagnosis of Alzheimer’s Disease | AD | AoR | sandeepcs07nta@gmail.com | India | 25 AD  25 ctrls |  |  | OCT | Unspecified | ^31^ |
| Optical coherence tomography reveals light-dependent retinal responses in Alzheimer’s disease | AD | AoR | bissig@ohsu.edu | USA | 14 AD  14 ctrls |  |  | OCT | Spectralis Heidelberg | ^32^ |
| Changes in retinal microvasculature and retinal layer thickness in association with apolipoprotein E genotype in Alzheimer’s disease | AD | AoR | minkim76@gmail.com | South Korea | 24 AD or MCI  31 ctrl |  | 77 | OCT  OCT-A | Zeiss- OCT-A  Spectralis Heidelberg- OCT | ^33^ |
| Macular Microvascular Density as a Diagnostic Biomarker for Alzheimer's Disease | AD | AoR | jbin0911@163.com | China | 77 AD  145 ctrls |  |  | OCT-A | Not specified in abstract | ^34^ |
| Evaluation of Ocular Perfusion in Alzheimer's Disease Using Optical Coherence Tomography Angiography | AD | AoR | maged.alnawaiseh@ukmuenster.de | Germany | 36 AD  38 Ctrls | 36 AD  38 Ctrls |  | OCT-A | RTVue XR Avanti | ^35^ |
| Is retinal vasculature a biomarker in amyloid proven Alzheimer's disease? | AD | AoR | j.denhaan1@vumc.nl | Netherlands | 48 AD  38 Ctrls |  |  | Fundus, OCT, OCT-A | Topcon TRC 50DX, Spectralis, Zeiss Model 5000 | ^36^ |
| Comparison of Retinal Microvasculature in Patients With Alzheimer's Disease and Primary Open-Angle Glaucoma by Optical Coherence Tomography Angiography | AD | AoR | przemo.zab@gmail.com | Poland | 27 AD  27 Ctrls |  |  | OCT-A | Optovue | ^37^ |
| Retinal microvascular attenuation in mental cognitive impairment and Alzheimer's disease by optical coherence tomography angiography | AD, MCI | AoR | fangliu_2004@yahoo.com | China | 18 AD  21 MCI  21 Ctrl | 28 AD  32 MCI  33 Ctrl |  | Fundus  OCT-A | Nidek  Optovue | ^38^ |
| Decreased Retinal Vascular Density in Alzheimer's Disease (AD) and Mild Cognitive Impairment (MCI): An Optical Coherence Tomography Angiography (OCTA) Study | AD, MCI | AoR | luzc@shmu.edu.cn | China | 62 AD  47 MCI  49 Ctrl | 62 AD  49 Ctrl |  | OCT-A | Optovue | ^39^ |
| Retinal microvasculature dysfunction is associated with Alzheimer's disease and mild cognitive impairment | AD, MCI | AoR | leopold.schmetterer@seri.com.sg | Singapore | 24 AD  37 MCI  39 Ctrl |  |  | OCT-A | Zeiss | ^40^ |
| A deep learning model for detection of Alzheimer's disease based on retinal photographs: a retrospective, multicentre case-control study | AD | AoR | carolcheung@cuhk.edu.hk | Hong Kong, Singapore, UK, USA | 688 AD  3200 ctrls |  | 12,949 | Fundus photo |  | ^41^ |
| Melanopsin retinal ganglion cell loss in Alzheimer disease | AD | AoR | chiaralamorgia@gmail.com | Italy | 21 AD |  |  | OCT | StratusOCT, Zeiss | ^42^ |
| Potential new diagnostic tool for Alzheimer's disease using a linear discriminant function for Fourier domain optical coherence tomography | AD | AoR | egmvivax@yahoo.com | Spain | 151 AD  61 Ctrl |  |  | OCT | Cirrus and Spectralis | ^43^ |
| Evaluation of retinal nerve fiber layer and ganglion cell layer thickness in Alzheimer's disease using spectral-domain optical coherence tomography | AD | AoR | giovanni.staurenghi@unimi.it | Italy | 21 AD  21 Ctrl |  |  | OCT | Spectralis and RTVue | ^44^ |
| Retinal microvascular network attenuation in Alzheimer’s disease | AD | AoR | g.j.mckay@qub.ac.uk | United Kingdom | 213 AD  294 ctrls |  |  | Fundus | 500 Canon CR-DGi | ^45^ |
| Microvascular network alterations in the retina of patients with Alzheimer's disease | AD | AoR | carolcheung@cuhk.edu.hk | Singapore | 136 AD  290 ctrls |  |  | Fundus | 45-degree digital  retinal camera (Canon CR-DGi 10D or Canon CR-1 40D;  Canon, Japan) | ^46^ |
| Retinal thickness in patients with mild cognitive impairment and Alzheimer's disease | AD, MCI | AoR | vedanicek@gmail.com | Israel | 30 AD  24 MCI  24 ctrls | 52 AD  40 MCI  38 ctrl |  | OCT | OCT3, Stratus, Carl Zeiss Meditec Inc., Dublin, CA, USA | ^47^ |
| Retinal nerve fiber layer thickness is associated with episodic memory deficit in mild cognitive impairment patients | MCI | AoR | kmsharonshen@gmail.com | China | 23 MCI  52 ctrls |  |  | OCT | Cirrus HD-OCT 4000 | ^48^ |
| Abnormal retinal thickness in patients with mild cognitive impairment and Alzheimer’s disease | AD, MCI | AoR | jacques.hugon@lrb.aphp.fr | France | 23 MCI  15 ctrls  26 AD |  |  | OCT | Stratus OCT3 | ^49^ |
| Retinal alterations in mild cognitive impairment and Alzheimer's disease: an optical coherence tomography study | AD, MCI | AoR | pmpjmp@gmail.com | Spain | 18 AD  21 MCI  41 ctrls |  |  | OCT | STRATUS OCT 3 (Carl Zeiss, Meditec Inc, USA) | ^50^ |
| Regularity changes of the retinal nerve fiber layer and macular ganglion cell complex in patients with the amnestic mild cognitive impairment | MCI | AoR | louiselu1964@163.com | China | 24 MCI  30 ctrls |  |  | OCT | RTVue (Optovue, Fremont, CA, USA) | ^51^ |
| Evaluation of inner retinal layers as biomarkers in mild cognitive impairment to moderate Alzheimer’s disease | AD, MCI | AoR | nora.lad@duke.edu | USA | 15 MCI  15 AD  18 ctrls | 30 MCI  30 AD  36 ctrls |  | SD-OCT | Spectralis | ^52^ |
| Retinal nerve Fiber layer measurement in patients with alzheimer’s disease | AD, MCI | AoR | Oční klinika LF UK a FN Plzeň | Czech Republic | 24 AD  10 MCI  26 ctrls | 48 AD  19 MCI  51 ctrls |  | OCT | Cirrus OCT | ^53^ |
| Retinal nerve fiber layer thickness in the patients with mild cognitive impairment or Alzheimer’s disease | AD, MCI | AoR | zxjune@gmail.com | China | 47 MCI  10 AD  167 ctrl |  |  | OCT | Spectralis | ^54^ |
| Usefulness of peripapillary nerve  fiber layer thickness assessed by  optical coherence tomography as a  biomarker for Alzheimer’s disease | AD, MCI | AoR | dsanchez@fundacioace.com | Spain | 192 MCI  324 AD  414 ctrls |  |  | OCT | Maestro Topcon | ^55^ |
| Functional and morphological  changes of the retinal vessels  in Alzheimer’s disease and mild  cognitive impairment | AD, MCI | AoR | giuseppe.querques@hotmail.it | Italy | 12 AD  12 MCI  12 Ctrls |  |  | OCT  OCT-A | Spectralis HRA OCT | ^56^ |
| Visualization of Focal Thinning of the Ganglion Cell–Inner Plexiform Layer in Patients with Mild Cognitive Impairment and Alzheimer’s Disease | AD, MCI | AoR | hjiang@med.miami.edu | USA | 25 AD  24 MCI  21 Ctrls |  |  | OCT | Custom Built UHR OCT device | ^57^ |
| Impaired retinal microcirculation in patients  with Alzheimer’s disease | AD, MCI | AoR | hjiang@med.miami.edu | USA | 20 AD  20 MCI  21 Ctrls |  |  | OCT | Zeiss Cirrus | ^58^ |
| Relationships between optic nerve damage and the severity of cognitive impairment in patients with mild cognitive impairment and Alzheimer’s disease. | AD, MCI | AoR | Yu Zhu  Department of Ophthalmology  The First Affiliated Hospital of Zhengzhou University  PR China | China | 49 MCI  46 AD  49 Ctrls |  |  | OCT | Stratus 3000, Carl Zeiss | ^59^ |
| Macular Thickness in Subjective Memory Complaints and Mild Cognitive Impairment: A Non-Invasive Biomarker | MCI | AoR | domingogimenez@gmail.com | Spain | 25 Ctrls  24 MCI | 50*  33 MCI |  | OCT | Cirrus HD | ^60^ |
| Retinal OCTA SEgmentation dataset (ROSE) | AD, MCI | Open Access | https://imed.nimte.ac.cn/dataofrose.html | China | 26 AD  13 ctrls |  | 229 | OCT-A | RTVue XR Avanti SD-OCT system (Optovue, USA) | ^61^ |
| Retinal microvascular and neurodegenerative changes in Alzheimer’s disease and mild cognitive impairment compared to controls | AD, MCI | AoR | sharon.fekrat@duke.edu | USA | 39 AD  37 MCI  133 ctrls | 70 AD  72 MCI 254 ctrls |  | OCT-A OCT | Zeiss Cirrus HD-5000 SD- OCT with AngioPlex OCTA | ^62^ |
| Altered macular microvasculature in mild cognitive impairment and Alzheimer disease | AD, MCI | AoR | hjiang@med.miami.edu | USA | 12 AD  19 MCI  21 controls |  |  | OCT, OCT-A | Zeiss | ^63^ |
| Choroidal Thickness in Patients with Mild Cognitive Impairment and Alzheimer's Type Dementia | AD, MCI | AoR | bulutme73@yahoo.com | Turkey | 41 AD  38 MCI  44 ctrls | 246 | 246 | EDI-OCT | Zeiss | ^64^ |
| Retinal Ganglion Cell Analysis Using High-Definition Optical Coherence Tomography in Patients with Mild Cognitive Impairment and Alzheimer's Disease | AD, MCI | AoR | carol.cheung.y.l@seri.com.sg | Taiwan | 100 AD  41 MCI  123 ctrls |  | 264 (100 AD, 41 MCI, 123 control) | SD-OCT | Zeiss | ^65^ |
| Macular Ganglion Cell -Inner Plexiform Layer Thickness Is Associated with Clinical Progression in Mild Cognitive Impairment and Alzheimer's Disease | AD, MCI | AoR | nrkim@inha.ac.kr | Korea | 42 AD  26 MCI  66 ctrls |  |  | OCT | Zeiss | ^66^ |
| Abnormal retinal nerve fiber layer thickness and macula lutea in patients with mild cognitive impairment and Alzheimer's disease | AD, MCI | AoR | yyliuping@163.com | China | 72 AD  26 MCI  21 ctrls |  |  | OCT | Cirrus HD-OCT | ^67^ |
| Retinal nerve fiber layer thickness in amnestic mild cognitive impairment: Case-control study and meta-analysis | AD, MCI | AoR | Amani A. Fawzi: moc.liamg@dmizwafa | USA | 17 MCI  17 ctrls |  |  | OCT  SD-OCT | Spectralis Heidelberg | ^68^ |
| Analysis of the Retinal Nerve Fiber Layer Thickness in Alzheimer Disease and Mild Cognitive Impairment | AD, MCI | AoR | Kimdk89@empas.com | Korea | 30 AD  30 MCI  30 ctrls |  |  | HD-OCT | Zeiss | ^69^ |
| The Association Between Retinal Neuronal Layer and Brain Structure is Disrupted in Patients with Cognitive Impairment and Alzheimer's Disease | AD, MCI | AoR | helen.zhou@duke-nus.edu.sg | Singapore | 47 AD  68 MCI  65 ctlrls |  |  | OCT | Unspecified | ^70^ |
| The relationship between the degree of cognitive impairment and retinal nerve fiber layer thickness | AD, MCI | AoR | ozdemir_ece@yahoo.com | Turkey | 35 AD  35 MCI  35 ctrl |  |  | OCT | Zeiss | ^71^ |
| Characterization of Inner Retinal Hyperreflective Alterations in Early Cognitive Impairment on Adaptive Optics Scanning Laser Ophthalmoscopy | AD, MCI | AoR | afawzimd@gmail.com | USA | 10 MCI  2 AD  14 control |  | 21 | OCT | Optovue | ^72^ |
| Retinal thinning in amyotrophic lateral sclerosis patients without ophthalmic disease | ALS | In article | Henry.Tseng@duke.edu | USA | 21 ALS |  |  | SD-OCT | Heidelberg Spectralis | ^73^ |
| Reduced retinal nerve fiber layer (RNFL) thickness in ALS patients: a window to disease progression | ALS | AoR | hajiakhoundi.f@iums.ac.ir | Iran | 20 ALS  25 controls |  |  | SD-OCT | Topcon 3D, 2015) | ^74^ |
| In vivo assessment of retinal vessel pathology in amyotrophic lateral sclerosis | ALS | AoR | elmar.pinkhardt@uni-ulm.de | Germany | 34 ALS  20 Ctrls |  |  | OCT | Spectralis | ^75^ |
| Changes in retinal OCT and their correlations with neurological disability in early ALS patients, a follow-up study | ALS | AoR | ramirezs@med.ucm.es | Spain | 10 ALS in first group  5 ALS follow-up  19 ctrls | 20/10 ALS  38 ctrls |  | OCT | Zeiss | ^76^ |
| Retinal involvement in amyotrophic lateral sclerosis: a study with optical coherence tomography and diffusion tensor imaging | ALS | AoR | elmar.pinkhardt@uni-ulm.de | Germany | 71 ALS  20 Ctrl |  |  | OCT | Spectralis | ^77^ |
| Comparison of optical coherence tomography findings and visual field changes in patients with primary open-angle glaucoma and amyotrophic lateral sclerosis | ALS | AoR | Puh3_ww@bjmu.edu.cn. | China | 51 ALS  126 ctrl |  |  | OCT | Zeiss Cirrus HD-OCT, Model 4000, Carl Zeiss Meditec, Inc. | ^78^ |
| Observing the brain through the eye: a longitudinal study on amyotrophic lateral sclerosis patients using optical coherence tomography and magnetic resonance imaging | ALS | AoR | andrei.marin@uni-ulm.de | Germany | 34 ALS  21 Ctrls |  |  | OCT | Spectralis | ^79^ |
| Retinal thinning in amyotrophic lateral sclerosis patients without ophthalmic disease | ALS | AoR | henry.tseng@duke.edu | USA | 21 ALS | 40 ALS |  | SD-OCT | Spectralis | ^73^ |
| Retinal involvement by optical coherence tomography and its correlation with disease severity in amyotrophic lateral sclerosis | ALS | AoR | unavailable | India | 25 ALS  25 Ctrl | 50 ALS  50 Ctrls |  | SD-OCT | Spectralis | ^80^ |
| Correlation between Retinal Vascularization and Disease Aggressiveness in Amyotrophic Lateral Sclerosis | ALS | AoR | xgilda@hotmail.com | Italy | 48 ALS  45 ctrls |  |  | OCT  OCT-A | Spectralis  Optovue Angiovue | ^81^ |
| Subtle retinal pathology in amyotrophic lateral sclerosis | ALS | AoR | orhan.aktas@uni-duesseldorf.de | Germany | 24 ALS  24 Ctrls | 48 ALS  48 Ctrls |  | OCT | Spectralis | ^82^ |
| Reduced retinal nerve fiber layer (RNFL) thickness in ALS patients: a window to disease progression | ALS | AoR | hajiakhoundi.f@iums.ac.ir | Iran | 20 ALS  25 Ctrls |  |  | OCT | Topcon 3D | ^74^ |
| Optical coherence tomography does not support optic nerve involvement in amyotrophic lateral sclerosis | ALS | AoR | friedemann.paul@charite.de | Germany | 76 ALS  54 Ctrls | 144 ALS  108 Ctrl | 498 total | OCT | Cirrus HD-OCT version 5.0 Zeiss | ^83^ |
| Macular sub-layer thinning and association with pulmonary function tests in Amyotrophic Lateral Sclerosis | ALS | AoR | afawzimd@gmail.com | US | 21 ALS  21 Ctrls |  |  | OCT | Spectralis | ^84^ |
| Development and validation of a deep learning algorithm based on fundus photographs for estimating the Cardiovascular Risk Factors, Aging, and Incidence of Dementia (CAIDE) dementia risk score | D-US | AoR | xiewuxiang@hsc.pku.edu.cn chenyuzhong@airdoc.com | China | 1,512 |  |  | Fundus Photographs | Unspecified | ^85^ |
| Optical Coherence Tomography Reveals Retinal Neuroaxonal Thinning in Frontotemporal Dementia as in Alzheimer’s Disease | D-US, AD, MCI, | AoR | letizia.leocani@hsr.it | Italy | 37 AD  29 MCI  49 ctrl |  |  | OCT | Information Inaccessible | ^86^ |
| Optical coherence tomography findings in Huntington’s disease: a potential biomarker of disease progression | HD | AoR | Richard H. Roxburgh richardr@adhb.govt.nz Hannah M. Kersten h.kersten@auckland.ac.n | New Zealand | 26 HD  29 ctrl |  |  | OCT | Heidelberg Spectralis | ^87^ |
| Huntington's disease and neurovascular structure of retina | HD | AoR | mohammadroohani@gmail.com | Iran | 25 HD  25 ctrls |  |  | SD-OCT  OCT-A | Spectralis AND  RTVue XR Optovue | ^88^ |
| Spectral-Domain Optical Coherence Tomography as a Potential Biomarker in Huntington's Disease | HD | AoR | carlos.hsjn@gmail.com | Portugal | 8 HD  8 ctrls | 15 HD  16 ctrl |  | SD-OCT | Spectralis | ^89^ |
| Optical coherence tomography (OCT) study in Argentinean Huntington's disease patients | HD | AoR | emiliamgatto@gmail.com | Argentina | 14 HD  13 ctrls | 27 HD  26 ctrl |  | SD-OCT | Spectralis Plus | ^90^ |
| Optical coherence tomography angiography findings in Huntington's disease | HD | AoR | da.montorio@gmail.com | Italy | 16 HD  13 ctrl | 32 HD  26 ctrl |  | SD-OCT, OCT-A | Spectralis AND Optovue RTVue | ^91^ |
| Retinal single-layer analysis with optical coherence tomography shows inner retinal layer thinning in Huntington's disease as a potential biomarker | HD | AoR | duygugsevim@gmail.com | Turkey | 15 HD  15 ctrls |  |  | OCT | Spectralis | ^92^ |
| No optical coherence tomography changes in premanifest Huntington's disease mutation carriers far from disease onset | Preclinical HD | AoR | anke.salmen@insel.ch | Switzerland | 24 HD  38 ctrls |  |  | SD-OCT | Spectralis | ^93^ |
| Structural Abnormalities of the Optic Nerve and Retina in Huntington's Disease Pre-Clinical and Clinical Settings | Preclinical HD | AoR | mielcarekml@gmail.com | Poland | 13 HD mutations, preclinical  14 ctrls |  |  | SD-OCT | Revo NX 110 (Optopol, Zawiercie, Poland | ^94^ |
| Retinal Imaging Techniques Based on Machine Learning Models in Recognition and Prediction of Mild Cognitive Impairment | MCI, D-US | AoR | zhangq226@mail.sysu.edu.cn huangdf@mail.sysu.edu.cn | China | 38 ctrls  26 MCI  22 D-US |  | 332 | Fundus | RetiCam 3100 | ^95^ |
| Electrophysiology and Optical Coherence Tomography to Evaluate Parkinson Disease Severity | PD | AoR | egmvivax@yahoo.com | Spain | 46 PD  33 Ctrls |  |  | OCT | Cirrus OCT | ^96^ |
| Development of a prediction formula of Parkinson disease severity by optical coherence tomography | PD | AoR | bea83jdm@hotmail.com | Spain | 52 PD  50 ctrils | 102 PD  97 ctrl |  | OCT | Spectralis | ^97^ |
| Retinal and Choroidal Changes in Patients with Parkinson's Disease Detected by Swept-Source Optical Coherence Tomography | PD | AoR | mariasatue@gmail.com | Spain | 50 PD  54 Ctrls | 50 PD  54 Ctrls |  | Swept-source OCT | DRI Triton SS-OCT device Topcon | ^98^ |
| CHARACTERIZATION BY FRACTAL DIMENSION ANALYSIS OF THE RETINAL CAPILLARY NETWORK IN PARKINSON DISEASE | PD | AoR | medhuaye@outlook.com. | China | 25 PD  25 Ctrls |  |  | OCT-A | Optovue RTVue XR Avanti | ^99^ |
| Retinal Microvascular Density Was Associated With the Clinical Progression of Parkinson’s Disease | PD | AoR | shenlu@csu.edu.cn | China | 115 PD  67 Ctrls |  |  | OCT-A | Cirrus 5000 HD-OCT by Zeiss | ^100^ |
| Retinal Microvascular Impairment in the Early Stages of Parkinson's Disease | PD | AoR | shenmxiao7@hotmail.com | China | 38 PD  28 ctrls | 49 PD  34 Ctrls |  | OCT-A | RTVue XR Avanti by Optovue | ^101^ |
| Visual Impairments Are Associated With Retinal Microvascular Density in Patients With Parkinson's Disease | PD | AoR | wanpengx@mail.sysu.edu.cn | China | 24 PD  23 Ctrls | 24 PD  23 Ctrls |  | OCT-A | Zeiss Cirrus HD-OCT 5000 with an AngioPlex OCTA instrument | ^102^ |
| Retinal Flow Density Changes in Early-stage Parkinson's Disease Investigated by Swept-Source Optical Coherence Tomography Angiography | PD | AoR | mingzhangscu@163.com | China | 42 PD  75 ctrls | 75 PD  150 ctrls |  | SS OCT  SS OCT-A | SVision commercial SSOCT system | ^103^ |
| Assessing Retinal Structure in Patients with Parkinson's Disease | PD | AoR | jcarroll@mcw.edu | USA | 33 PD  40 ctrls |  |  | SD-OCT | CirrusTM Zeiss | ^104^ |
| Inner retinal layer thinning in Parkinson disease | PD | AoR | ivan.bodis-wollner@downstate.edu | USA | 24 PD  17 Ctrl | 45 PD  31 Ctrl |  | OCT | RTvue, Optovue | ^105^ |
| Ability and Reproducibility of FourierDomain Optical Coherence Tomography to Detect Retinal Nerve Fiber Layer Atrophy  in Parkinson’s Disease | PD | AoR | egmvivax@yahoo.com | Spain | 75 PD  75 Ctrls |  |  | FD-OCT | Cirrus AND Spectralis | ^96^ |
| Optical Coherence Tomography in Parkinsonian  Syndromes | PD | AoR | phil.albrecht@gmail.com | Germany | PD 40  35 ctrls |  |  | SD-OCT | Spectralis | ^106^ |
| Use of Fourier-domain OCT to detect retinal nerve fiber layer degeneration in Parkinson’s disease patients | PD | AoR | egmvivax@yahoo.com | Spain | 100 PD  100 Ctrls |  |  | FD-OCT | Cirrus AND Spectralis | ^107^ |
| Retinal nerve changes in patients with tremor dominant and akinetic rigid Parkinson's disease | PD | AoR | shadighurchian@gmail.com | Iran | 27 PD  25 Ctrl |  |  | 3D-OCT | 3d- oCT 1000 Topcon | ^108^ |
| Retinal Nerve Fiber Layer Thickness in Parkinson Disease | PD | AoR | drskirbas@gmail.com | Turkey | 42 PD  40 Ctrl |  |  | OCT | Cirrus | ^109^ |
| Effects of Levodopa on Retina in Parkinson Disease | PD | AoR | aysehir@yahoo.com | Turkey | 35 PD  11 Ctrl |  |  | OCT | Cirrus | ^110^ |
| Distribution of Retinal Layer Atrophy in Patients With Parkinson Disease and Association With Disease Severity and Duration | PD | AoR | egmvivax@yahoo.com | Spain | 129 PD  129 Ctrls |  |  | OCT | Spectralis | ^111^ |
| RETINA MEASUREMENTS FOR DIAGNOSIS OF PARKINSON DISEASE | PD | AoR | egmvivax@yahoo.com | Spain | 111 PD  200 Ctrls |  |  | OCT | Spectralis | ^112^ |
| Ganglion Cell–Inner Plexiform Layer Thickness in Patients With Parkinson Disease and Association With Disease Severity and Duration | PD | AoR | dresinsogutlu@gmail.com | Turkey | 54 PD  54 Ctrls |  |  | OCT | Cirrus | ^113^ |
| The Association of Spectral-Domain Optical Coherence Tomography Determined Ganglion Cell Complex Parameters and Disease Severity in Parkinson’s Disease | PD | AoR | alihasanbayhan@hotmail.com | Turkey | 20 PD  30 Ctrls |  |  | OCT | RTVue | ^114^ |
| Retinal nerve fiber layer thickness and visual hallucinations in Parkinson's Disease | PD | AoR | twkim93@medimail.co.kr | Republic of Korea | 56 PD  30 Ctrls |  |  | OCT | Opko | ^115^ |
| Applications of visual evoked potentials and Fourier-domain optical coherence tomography in Parkinson's disease: a controlled study | PD | AoR | lucasquagliato@yahoo.com.br | Brazil | 43 PD  38 Ctrl |  |  | OCT | RTVue | ^116^ |
| Retinal thinning and correlation with functional disability in patients with Parkinson’s Disease | PD | AoR | mariasatue@gmail.com | Spain | 153 PD  242 Ctrl |  |  | OCT | Spectralis | ^117^ |
| Retinal single-layer analysis in Parkinsonian syndromes: an optical coherence tomography study | PD | AoR | elmar.pinkhardt@uni-ulm.de | Germany | 65 PD  41 Ctrl |  |  | OCT | Cirrus | ^118^ |
| Photoreceptor layer thinning in idiopathic Parkinson's disease | PD | AoR | friedemann.paul@charite.de | Germany | 68 PD  32 Ctrl |  |  | OCT | Cirrus | ^119^ |
| Scanning laser polarimetry and spectral domain optical coherence tomography for the detection of retinal changes in Parkinson's disease | PD | AoR | b.stemplewitz@uke.de | Germany | 108 PD  165 Ctrl |  |  | OCT | Cirrus | ^120^ |
| Detection of retinal changes in idiopathic Parkinson's disease using high-resolution optical coherence tomography and heidelberg retina tomography | PD | AoR | d.bittersohl@uke.de | Germany | 108 PD  165 Ctrl |  |  | OCT | Spectralis | ^121^ |
| Correlation between structural and functional retinal changes in Parkinson disease: | PD | AoR | rohitsaxena80@yahoo.com | India | 20 PD  20 Ctrl |  |  | OCT | Cirrus | ^122^ |
| Optical coherence tomography as a tool to evaluate retinal changes in Parkinson's disease | PD | AoR | pal.pramod@rediffmail.com | India | 30 PD  30 Ctrl |  |  | OCT | Spectralis | ^123^ |
| In vivo morphology of the optic nerve and retina in patients with Parkinson's disease | PD | AoR | ig15@le.ac.uk | United Kingdom | 25 PD  25 Ctrl |  |  | OCT | Copernicus | ^124^ |
| Comparison of optical coherence tomography findings in patients with primary open-angle glaucoma and Parkinson disease | PD | AoR | muhsineraslan@hotmail.com | Turkey | 25 PD  23 Ctrl |  |  | OCT | RTVue | ^125^ |
| Analysis of the retinal nerve fiber and ganglion cell – inner plexiform layer by optical coherence tomography in Parkinson's patients | PD | AoR | turgayucak10@gmail.com | Turkey | 30 PD  30 Ctrl |  |  | OCT | Cirrus | ^126^ |
| Visual dysfunction and its correlation with retinal changes in patients with Parkinson's disease: an observational cross-sectional study | PD | AoR | mariasatue@gmail.com | Spain | 37 PD  37 Ctrl |  |  | OCT | Cirrus | ^127^ |
| Evaluation of progressive visual dysfunction and retinal degeneration in patients with Parkinson's disease | PD | AoR | mariasatue@gmail.com | Spain | 30 PD  30 Ctrl |  |  | OCT | Spectralis | ^128^ |
| Evaluation of Retinal Changes in Progressive Supranuclear Palsy and Parkinson Disease | PD | AoR | duygugsevim@gmail.com | Turkey | 29 PD  33 Ctrl |  |  | SD-OCT | Spectralis | ^129^ |
| Retinal vessel diameter obtained by optical coherence tomography is spared in Parkinson’s disease | PD | AoR | duygugsevim@gmail.com | Turkey | 41 PD  35 Ctrl |  |  | SD-OCT | Spectralis | ^130^ |
| In vivo exploration of retinal nerve fiber layer morphology in Parkinson's disease patients | PD | AoR | visser.femke@gmail.com | Netherlands | 20 PD  20 Ctrl |  |  | OCT | Spectralis | ^131^ |
| Combination of multifocal electroretinogram and spectral-domain OCT can increase diagnostic efficacy of Parkinson's disease | PD | AoR | luoweifengsz@sohu.com | China | 53 PD  41 Ctrl |  |  | OCT | Cirrus | ^132^ |
| Retinal changes in Parkinson's disease and glaucoma | PD | AoR | juliane.matlach@unimedizin-mainz.de | Germany | 40 PD  23 Ctrl |  |  | OCT | Cirrus | ^133^ |
| Progressive changes in the retinal structure of patients with Parkinson's disease | PD | AoR | liuchunfeng@suda.edu.cn | China | 37 PD  42 Ctrl |  |  | OCT | Cirrus | ^134^ |
| Evaluation of choroidal and retinal thickness changes in Parkinson's disease using spectral domain optical coherence tomography | PD | AoR | eirchat@yahoo.gr | Greece | 31 PD  25 Ctrl |  |  | OCT | Spectralis | ^135^ |
| Correlations among multifocal electroretinography and optical coherence tomography findings in patients with Parkinson's disease | PD | AoR | drunlumetin@hotmail.com | Turkey | 58 PD  30 Ctrl |  |  | OCT | Spectralis | ^136^ |
| Optical coherence tomography findings in Parkinson's disease | PD | AoR | dr.mogun@gmail.com | Turkey | 25 PD  29 Ctrl |  |  | OCT | Spectralis | ^137^ |
| Evaluation of retinal alterations in Parkinson disease and tremor diseases | PD | AoR | betultugcu@gmail.com | Turkey | 33 PD  22 Ctrls | 64 PD  44 Ctrls |  | OCT | Information Inaccessible | ^138^ |
| Perimetric and retinal nerve fiber layer findings in patients with Parkinson's disease | PD | AoR | e_tsironi@hotmail.com | Greece | 24 PD  24 Ctrls | 24 PD  24 Ctrls |  | OCT | Stratus OCT, Zeiss | ^139^ |
| Optical coherence tomography of patients with Parkinson’s disease and progressive supranuclear palsy | PD | AoR | samir.alkabie@downstate.edu | USA | 12 PD  12 Ctrl |  |  | OCT | Spectralis | ^140^ |
| Retinal thickness in Parkinson's disease | PD | AoR | neilarchie@me.com | United Kingdom | 51 PD  25 Ctrl |  |  | OCT | Zeiss Stratus 3000 | ^141^ |
| Combination of optical coherence tomography (OCT) and OCT angiography increases diagnostic efficacy of Parkinson's disease | PD | AoR | xhz1030@csu.edu.cn | China | 35 PD  35 Ctrl | 35 PD  35 Ctrl |  | OCT-A | Cirrus Zeiss | ^142^ |
| Characterization of Retinal Microvascular and Choroidal Structural Changes in Parkinson Disease | PD | AoR | dilraj.grewal@duke.edu | USA | 69 PD  137 Ctrl | 124 PD  248 Ctrl |  | OCT-A | Zeiss Cirrus | ^143^ |
| Retinal Thickness Predicts the Risk of Cognitive Decline in Parkinson Disease | PD | AoR | ane.muruetagoyena@osakidetza.eus | Spain | 49 PD  40 Ctrl | 87 PD  73 Ctrl |  | OCT-A | Spectralis | ^144^ |
| Pilot study for neurological and retinal imaging as biomarkers for Parkinson's disease using optical coherence tomography-angiography | PD | Not yet available | N/A | USA | 7 PD |  |  | OCT-A | RTVue | ^145^ |
| Retinal Thickness and Microvascular Pattern in Early Parkinson's Disease | PD | AoR | anicolet@unict.it | Italy | 21 PD  17 Ctrl | 41 PD  33 Ctrls |  | SD-OCT  OCT-A | Cirrus Zeiss | ^146^ |
| Retinal texture biomarkers may help to discriminate between Alzheimer’s, Parkinson’s, and healthy control | PD, AD | AoR | rmbernardes@fmed.uc.pt | Portugal | 20 AD  28 PD | 27 Ctrls |  | OCT | Zeiss | ^147^ |
| Retinal Nerve Fiber Layer Thinning in Alzheimer's Disease: A Case-Control Study in Comparison to Normal Aging, Parkinson's Disease, and Non-Alzheimer's Dementia | PD, AD, MCI | AoR | pillaij@ccf.org | USA | 21 AD  20 MCI  20 D-US  20 PD  34 ctrl |  |  | SD-OCT  HD-OCT | Zeiss | ^148^ |
| Association of Preclinical Alzheimer Disease With Optical Coherence Tomographic Angiography Findings | Preclinical AD | AoR | apte@wustl.edu vantaverng@wustl.edu | USA | 30 | 58 |  | OCTA | Avanti Optovue | ^149^ |
| Nonvascular retinal imaging markers of preclinical Alzheimer's disease | Preclinical AD | AoR | psnyder@lifespan.org | USA | 63 Preclinical AD |  |  | SD-OCT | Spectralis | ^150^ |
| Retinal nerve fiber layer thickness predicts CSF amyloid/tau before cognitive decline | Preclinical AD | AoR | In article | USA | 27 pathological  16 clear |  |  | SD- OCT | Zeiss | ^151^ |
| Machine-learning method for localization of cerebral white matter hyperintensities in healthy adults based on retinal images | white matter disease, unspecified | AoR | bzee@cuhk.edu.hk | Hong Kong | 240 |  |  | Fundus photograph | CR-2 AF, Canon Singapore  Topcon, Japan | ^152^ |

1. Lustig-Barzelay Y, Sher I, Sharvit-Ginon I, et al. Machine learning for comprehensive prediction of high risk for Alzheimer’s disease based on chromatic pupilloperimetry. *Sci Rep*. 2022;12:9945. doi:10.1038/s41598-022-13999-0

2. Wisely CE, Wang D, Henao R, et al. Convolutional neural network to identify symptomatic Alzheimer’s disease using multimodal retinal imaging. *Br J Ophthalmol*. 2022;106(3):388-395. doi:10.1136/bjophthalmol-2020-317659

3. Marquié M, Valero S, Castilla-Marti M, et al. Association between retinal thickness and β-amyloid brain accumulation in individuals with subjective cognitive decline: Fundació ACE Healthy Brain Initiative. *Alzheimers Res Ther*. 2020;12(1):37. doi:10.1186/s13195-020-00602-9

4. Tian J, Smith G, Guo H, et al. Modular machine learning for Alzheimer’s disease classification from retinal vasculature. *Sci Rep*. 2021;11:238. doi:10.1038/s41598-020-80312-2

5. JA K, HT N, E K. Longitudinal retinal layer changes in preclinical Alzheimer’s disease. *Acta Ophthalmol Copenh*. 2021;99(5):538-544. doi:10.1111/aos.14640

6. López-de-Eguileta A, Lage C, López-García S. Evaluation of choroidal thickness in prodromal Alzheimer’s disease defined by amyloid PET. *PLOS ONE*. 2020;15(9). doi:10.1371/journal.pone.0239484

7. Bulut M, Kurtuluş F, Gözkaya O. Evaluation of optical coherence tomography angiographic findings in Alzheimer’s type dementia. *Br J Ophthalmol*. 2018;102(2):233-237. doi:10.1136/bjophthalmol-2017-310476

8. Koronyo Y, Biggs D, Barron E. Retinal amyloid pathology and proof-of-concept imaging trial in Alzheimer’s disease. *JCI Insight*. 2(16). doi:10.1172/jci.insight.93621

9. Corbin D, Lesage F. Assessment of the predictive potential of cognitive scores from retinal images and retinal fundus metadata via deep learning using the CLSA database. *Sci Rep*. 2022;12(1). doi:10.1038/s41598-022-09719-3

10. Bambo MP, Garcia-Martin E, Gutierrez-Ruiz F. Analysis of optic disk color changes in Alzheimer’s disease: A potential new biomarker. *Clin Neurol Neurosurg*. 2015;132:68-73. doi:10.1016/j.clineuro.2015.02.016

11. Bayhan HA, Aslan Bayhan S, Celikbilek A, Tanık N, Gürdal C. Evaluation of the chorioretinal thickness changes in Alzheimer’s disease using spectral-domain optical coherence tomography. *Clin Exp Ophthalmol*. 2015;43(2):145-151. doi:10.1111/ceo.12386

12. Cunha LP, Lopes LC, Costa-Cunha LVF. Macular Thickness Measurements with Frequency Domain-OCT for Quantification of Retinal Neural Loss and its Correlation with Cognitive Impairment in Alzheimerʼs Disease. *PLoS ONE*. 2016;11(4). doi:10.1371/journal.pone.0153830

13. Cunha JP, Proença R, Dias-Santos A. OCT in Alzheimer’s disease: thinning of the RNFL and superior hemiretina. *Graefes Arch Clin Exp Ophthalmol*. 2017;255(9):1827-1835. doi:10.1007/s00417-017-3715-9

14. Cunha JP, Proença R, Dias-Santos A. Choroidal thinning: Alzheimer’s disease and aging. *Alzheimers Dement Diagn Assess Monit*. 2017;8:11-17. doi:10.1016/j.dadm.2017.03.004

15. Eraslan M, Çerman E, Çeki̇ç O. Neurodegeneration in ocular and central nervous systems: optical coherence tomography study in normal-tension glaucoma and Alzheimer disease. *Turk J Med Sci*. 2015;45(5):1106-1114. doi:10.3906/sag-1406-145

16. Garcia-Martin E, Bambo MP, Marques ML. Ganglion cell layer measurements correlate with disease severity in patients with Alzheimer’s disease. *Acta Ophthalmol Copenh*. 2016;94(6). doi:10.1111/aos.12977

17. Gharbiya M, Trebbastoni A, Parisi F. Choroidal Thinning as a New Finding in Alzheimer’s Disease: Evidence from Enhanced Depth Imaging Spectral Domain Optical Coherence Tomography. *J Alzheimers Dis*. 2014;40(4):907-917. doi:10.3233/JAD-132039

18. Golzan SM, Goozee K, Georgevsky D. Retinal vascular and structural changes are associated with amyloid burden in the elderly: ophthalmic biomarkers of preclinical Alzheimer’s disease. *Alzheimers Res Ther*. 2017;9(13). doi:10.1186/s13195-017-0239-9

19. Güneş A, Demirci S, Tök L, Tök Ö, Demirci S. Evaluation of retinal nerve fiber layer thickness in Alzheimer disease using spectral-domain optical coherence tomography. *Turk J Med Sci*. 2015;45(5):1094-1097.

20. Kirbas S, Turkyilmaz K, Anlar O, Tufekci A, Durmus M. Retinal nerve fiber layer thickness in patients with Alzheimer disease. *J Neuro-Ophthalmol Off J North Am Neuro-Ophthalmol Soc*. 2013;33(1):58-61. doi:10.1097/WNO.0b013e318267fd5f

21. Kromer R, Serbecic N, Hausner L, Froelich L, Aboul-Enein F, Beutelspacher SC. Detection of Retinal Nerve Fiber Layer Defects in Alzheimer’s Disease Using SD-OCT. *Front Psychiatry*. 2014;5(22). doi:10.3389/fpsyt.2014.00022

22. Larrosa JM, Garcia-Martin E, Bambo MP. Potential New Diagnostic Tool for Alzheimer’s Disease Using a Linear Discriminant Function for Fourier Domain Optical Coherence Tomography. *Invest Ophthalmol Vis Sci*. 2014;55(5):3043-3051. doi:10.1167/iovs.13-13629

23. Güneş A, Demirci S, Tök L, Tök Ö, Demirci S. Evaluation of retinal nerve fiber layer thickness in Alzheimer disease using spectral-domain optical coherence tomography. *Turk J Med Sci*. 2015;45(5):1094-1097.

24. Moreno-Ramos T, Benito-León J, Villarejo A, Bermejo-Pareja F. Retinal nerve fiber layer thinning in dementia associated with Parkinson’s disease, dementia with Lewy bodies, and Alzheimer’s disease. *J Alzheimers JAD*. 2013;34(3):659-664. doi:10.3233/JAD-121975

25. Polo V, Garcia-Martin E, Bambo MP. Reliability and validity of Cirrus and Spectralis optical coherence tomography for detecting retinal atrophy in Alzheimer’s disease. *Eye Lond Engl*. 2014;28(6):680-690. doi:10.1038/eye.2014.51

26. Polo V, Rodrigo MJ, Garcia-Martin E. Visual dysfunction and its correlation with retinal changes in patients with Alzheimer’s disease. *Eye Lond Engl*. 2017;31(7):1034-1041. doi:10.1038/eye.2017.23

27. Salobrar-Garcia E, Hoyas I, Leal M. Analysis of Retinal Peripapillary Segmentation in Early Alzheimer’s Disease Patients. *BioMed Res Int*. 2015;2015(636548). doi:10.1155/2015/636548

28. Trebbastoni A, D’Antonio F, Bruscolini A. Retinal nerve fibre layer thickness changes in Alzheimer’s disease: Results from a 12-month prospective case series. *Neurosci Lett*. 2016;629:165-170. doi:10.1016/j.neulet.2016.07.006

29. T A, M M, M F. Attenuation of Choroidal Thickness in Patients With Alzheimer Disease: Evidence From an Italian Prospective Study. *Alzheimer Assoc Disord*. 2017;31(2). doi:10.1097/WAD.0000000000000176

30. Lemmens S, Craenendonck T, Eijgen J. Combination of snapshot hyperspectral retinal imaging and optical coherence tomography to identify Alzheimer’s disease patients. *Alzheimers Res Ther*. 2020;12(1). doi:10.1186/s13195-020-00715-1

31. Sandeep CS, Sukesh Kumar A, Mahadevan K, Manoj P. Analysis of Retinal OCT Images for the Early Diagnosis of Alzheimer’s Disease. In: Chattopadhyay S, Roy T, Sengupta S, Berger-Vachon C, eds. *Modelling and Simulation in Science, Technology and Engineering Mathematics. Advances in Intelligent Systems and Computing*. Springer International Publishing; 2019. doi:10.1007/978-3-319-74808-5_43

32. Bissig D, Zhou CG, Le V, Bernard JT. Optical coherence tomography reveals light-dependent retinal responses in Alzheimer’s disease. *NeuroImage*. 2020;219(117022). doi:10.1016/j.neuroimage.2020.117022

33. Shin JY, Choi EY, Kim M, Lee HK, Byeon SH. Changes in retinal microvasculature and retinal layer thickness in association with apolipoprotein E genotype in Alzheimer’s disease. *Sci Rep*. 2021;11(1). doi:10.1038/s41598-020-80892-z

34. Wang X, Wang Y, Liu H, et al. Macular Microvascular Density as a Diagnostic Biomarker for Alzheimer’s Disease. *J Alzheimers Dis JAD*. 2022;90(1):139-149. doi:10.3233/JAD-220482

35. Lahme L, Esser EL, Mihailovic N, et al. Evaluation of Ocular Perfusion in Alzheimer’s Disease Using Optical Coherence Tomography Angiography. *J Alzheimers Dis JAD*. 2018;66(4):1745-1752. doi:10.3233/JAD-180738

36. den Haan J, van de Kreeke JA, van Berckel BN, et al. Is retinal vasculature a biomarker in amyloid proven Alzheimer’s disease? *Alzheimers Dement Amst Neth*. 2019;11:383-391. doi:10.1016/j.dadm.2019.03.006

37. Zabel P, Kaluzny JJ, Wilkosc-Debczynska M, et al. Comparison of Retinal Microvasculature in Patients With Alzheimer’s Disease and Primary Open-Angle Glaucoma by Optical Coherence Tomography Angiography. *Invest Ophthalmol Vis Sci*. 2019;60(10):3447-3455. doi:10.1167/iovs.19-27028

38. Wu J, Zhang X, Azhati G, Li T, Xu G, Liu F. Retinal microvascular attenuation in mental cognitive impairment and Alzheimer’s disease by optical coherence tomography angiography. *Acta Ophthalmol (Copenh)*. 2020;98(6):e781-e787. doi:10.1111/aos.14381

39. Wang X, Zhao Q, Tao R, et al. Decreased Retinal Vascular Density in Alzheimer’s Disease (AD) and Mild Cognitive Impairment (MCI): An Optical Coherence Tomography Angiography (OCTA) Study. *Front Aging Neurosci*. 2020;12:572484. doi:10.3389/fnagi.2020.572484

40. Chua J, Hu Q, Ke M, et al. Retinal microvasculature dysfunction is associated with Alzheimer’s disease and mild cognitive impairment. *Alzheimers Res Ther*. 2020;12(1):161. doi:10.1186/s13195-020-00724-0

41. Cheung CY, Ran AR, Wang S. A deep learning model for detection of Alzheimer’s disease based on retinal photographs: a retrospective, multicentre case-control study. *Lancet Digit Health*. 2022;4(11). doi:10.1016/S2589-7500(22)00169-8

42. La Morgia C, Ross‐Cisneros FN, Koronyo Y, et al. Melanopsin retinal ganglion cell loss in Alzheimer disease. *Ann Neurol*. 2016;79(1):90-109. doi:10.1002/ana.24548

43. Larrosa JM, Garcia-Martin E, Bambo MP, et al. Potential new diagnostic tool for Alzheimer’s disease using a linear discriminant function for Fourier domain optical coherence tomography. *Invest Ophthalmol Vis Sci*. 2014;55(5):3043-3051. doi:10.1167/iovs.13-13629

44. Marziani E, Pomati S, Ramolfo P, et al. Evaluation of retinal nerve fiber layer and ganglion cell layer thickness in Alzheimer’s disease using spectral-domain optical coherence tomography. *Invest Ophthalmol Vis Sci*. 2013;54(9):5953-5958. doi:10.1167/iovs.13-12046

45. Williams MA, McGowan AJ, Cardwell CR, et al. Retinal microvascular network attenuation in Alzheimer’s disease. *Alzheimers Dement Amst Neth*. 2015;1(2):229-235. doi:10.1016/j.dadm.2015.04.001

46. Cheung CYL, Ong YT, Ikram MK, et al. Microvascular network alterations in the retina of patients with Alzheimer’s disease. *Alzheimers Dement J Alzheimers Assoc*. 2014;10(2):135-142. doi:10.1016/j.jalz.2013.06.009

47. Kesler A, Vakhapova V, Korczyn AD, Naftaliev E, Neudorfer M. Retinal thickness in patients with mild cognitive impairment and Alzheimer’s disease. *Clin Neurol Neurosurg*. 2011;113(7):523-526. doi:10.1016/j.clineuro.2011.02.014

48. Shen Y, Liu L, Cheng Y, et al. Retinal nerve fiber layer thickness is associated with episodic memory deficit in mild cognitive impairment patients. *Curr Alzheimer Res*. 2014;11(3):259-266. doi:10.2174/1567205011666140131114418

49. Paquet C, Boissonnot M, Roger F, Dighiero P, Gil R, Hugon J. Abnormal retinal thickness in patients with mild cognitive impairment and Alzheimer’s disease. *Neurosci Lett*. 2007;420(2):97-99. doi:10.1016/j.neulet.2007.02.090

50. Ascaso FJ, Cruz N, Modrego PJ, et al. Retinal alterations in mild cognitive impairment and Alzheimer’s disease: an optical coherence tomography study. *J Neurol*. 2014;261(8):1522-1530. doi:10.1007/s00415-014-7374-z

51. Wu Y, Wang XN, Wang N, Han Y, Ma D, Lu Y. Regularity changes of the retinal nerve fiber layer and macular ganglion cell complex in patients with the amnestic mild cognitive impairment. *Int J Neurosci*. 2018;128(9):849-853. doi:10.1080/00207454.2018.1438428

52. Lad EM, Mukherjee D, Stinnett SS, et al. Evaluation of inner retinal layers as biomarkers in mild cognitive impairment to moderate Alzheimer’s disease. *PLOS ONE*. 2018;13(2):e0192646. doi:10.1371/journal.pone.0192646

53. Kasl Z, Rusňák Š, Jirásková N. Retinal Nerve Fiber Layer Measurement in Patients with Alzheimer’s Disease. *Čes Slov Neurol Neurochir*. 2016;79/112(4):424-429. doi:10.14735/amcsnn2016424

54. Zhu LP, Ren X lei, Wang Y xing, Xu L, Zhang, Xiao-jun. Retinal nerve fiber layer thickness in the patients with mild cognitive impairment or Alzheimer’s disease. *Ophthalmol China*. 2014;23(4):231. doi:10.13281/j.cnki.issn.1004-4469.2014.04.004

55. Sánchez D, Castilla-Marti M, Rodríguez-Gómez O, et al. Usefulness of peripapillary nerve fiber layer thickness assessed by optical coherence tomography as a biomarker for Alzheimer’s disease. *Sci Rep*. 2018;8(1):16345. doi:10.1038/s41598-018-34577-3

56. Querques G, Borrelli E, Sacconi R, et al. Functional and morphological changes of the retinal vessels in Alzheimer’s disease and mild cognitive impairment. *Sci Rep*. 2019;9(1):63. doi:10.1038/s41598-018-37271-6

57. Shao Y, Jiang H, Wei Y, et al. Visualization of focal thinning of the ganglion cell–inner plexiform layer in patients with mild cognitive impairment and Alzheimer’s disease. *J Alzheimers Dis*. 2018;64(4):1261-1273.

58. Jiang H, Liu Y, Wei Y, et al. Impaired retinal microcirculation in patients with Alzheimer’s disease. Mogi M, ed. *PLOS ONE*. 2018;13(2):e0192154. doi:10.1371/journal.pone.0192154

59. Zhang L, Xu Y, Zhu Y. Relationships between optic nerve damage and the severity of cognitive impairment in patients with mild cognitive impairment and Alzheimer’s disease. *Biomed Res 0970-938X*. 2017;28(13).

60. Giménez Castejón D, Dudekova M, Gómez Gallego M, Lajara Blesa J. Macular Thickness in Subjective Memory Complaints and Mild Cognitive Impairment: A Non-Invasive Biomarker. *Neuro-Ophthalmol*. 2016;40(1):16-22. doi:10.3109/01658107.2015.1118516

61. Ma Y, Hao H, Xie J. ROSE: A Retinal OCT-Angiography Vessel Segmentation Dataset and New Model. *IEEE Trans Med Imaging*. 2021;40(3):928-939. doi:10.1109/TMI.2020.3042802

62. Yoon SP, Grewal DS, Thompson AC. Retinal microvascular and neurodegenerative changes in Alzheimer’s disease and mild cognitive impairment compared to controls. *Ophthalmol Retina*. 2019;3(6):489-499. doi:10.1016/j.oret.2019.02.002

63. Jiang H, Wei Y, Shi Y. Altered macular microvasculature in mild cognitive impairment and Alzheimer disease. *J Neuro-Ophthalmol J North Am Neuro-Ophthalmol Soc*. 2018;38(3):292-298. doi:10.1097/WNO.0000000000000580

64. Bulut M, Yaman A, Erol MK. Choroidal Thickness in Patients with Mild Cognitive Impairment and Alzheimer’s Type Dementia. *J Ophthalmol*. 2016;2016(7291257). doi:10.1155/2016/7291257

65. Cheung CY, Wong WLE, Hilal S, et al. Deep-learning retinal vessel calibre measurements and risk of cognitive decline and dementia. *Brain Commun*. 2022;4(4):fcac212. doi:10.1093/braincomms/fcac212

66. Choi SH, Park SJ, Kim NR. Macular Ganglion Cell -Inner Plexiform Layer Thickness Is Associated with Clinical Progression in Mild Cognitive Impairment and Alzheimers Disease. *PloS One*. 2016;11(9). doi:10.1371/journal.pone.0162202

67. Gao L, Liu Y, Li X, Bai Q, Liu P. Abnormal retinal nerve fiber layer thickness and macula lutea in patients with mild cognitive impairment and Alzheimer’s disease. *Arch Gerontol Geriatr*. 2015;60(1):162-167. doi:10.1016/j.archger.2014.10.011

68. Knoll B, Simonett J, Volpe NJ. Retinal nerve fiber layer thickness in amnestic mild cognitive impairment: Case-control study and meta-analysis. *Alzheimers Dement Amst Neth*. 2016;4:85-93. doi:10.1016/j.dadm.2016.07.004

69. Kwon JY, Yang JH, Han JS, Kim DG. Analysis of the Retinal Nerve Fiber Layer Thickness in Alzheimer Disease and Mild Cognitive Impairment. *Korean J Ophthalmol KJO*. 2017;31(6):548-556. doi:10.3341/kjo.2016.0118

70. Liu S, Ong YT, Hilal S. The Association Between Retinal Neuronal Layer and Brain Structure is Disrupted in Patients with Cognitive Impairment and Alzheimer’s Disease. *J Alzheimers JAD*. 2016;54(2):585-595. doi:10.3233/JAD-160067

71. Oktem EO, Derle E, Kibaroglu S, Oktem C, Akkoyun I, Can U. The relationship between the degree of cognitive impairment and retinal nerve fiber layer thickness. *Neurol Sci J Ital Neurol Soc Ital Soc Clin Neurophysiol*. 2015;36(7):1141-1146. doi:10.1007/s10072-014-2055-3

72. Zhang YS, Onishi AC, Zhou N. Characterization of Inner Retinal Hyperreflective Alterations in Early Cognitive Impairment on Adaptive Optics Scanning Laser Ophthalmoscopy. *Invest Ophthalmol Vis Sci*. 2019;60(10):3527-3536. doi:10.1167/iovs.19-27135

73. Mukherjee N, McBurney-Lin S, Kuo A, Bedlack R, Tseng H. Retinal thinning in amyotrophic lateral sclerosis patients without ophthalmic disease. *PLoS ONE*. 2017;12(9). doi:10.1371/journal.pone.0185242

74. Rohani M, Meysamie A, Zamani B, Sowlat MM, Akhoundi FH. Reduced retinal nerve fiber layer (RNFL) thickness in ALS patients: a window to disease progression. *J Neurol*. 2018;265(7):1557-1562. doi:10.1007/s00415-018-8863-2

75. Abdelhak A, Hübers A, Böhm K, Ludolph AC, Kassubek J, Pinkhardt EH. In vivo assessment of retinal vessel pathology in amyotrophic lateral sclerosis. *J Neurol*. 2018;265(4):949-953. doi:10.1007/s00415-018-8787-x

76. Rojas P, de Hoz R, Ramírez AI, et al. Changes in Retinal OCT and Their Correlations with Neurological Disability in Early ALS Patients, a Follow-Up Study. *Brain Sci*. 2019;9(12):337. doi:10.3390/brainsci9120337

77. Hübers A, Müller HP, Dreyhaupt J, et al. Retinal involvement in amyotrophic lateral sclerosis: a study with optical coherence tomography and diffusion tensor imaging. *J Neural Transm Vienna Austria 1996*. 2016;123(3):281-287. doi:10.1007/s00702-015-1483-4

78. Liu Z, Wang H, Fan D, Wang W. Comparison of optical coherence tomography findings and visual field changes in patients with primary open-angle glaucoma and amyotrophic lateral sclerosis. *J Clin Neurosci Off J Neurosurg Soc Australas*. 2018;48:233-237. doi:10.1016/j.jocn.2017.10.080

79. Marin AV. *Observing the Brain through the Eye: A Longitudinal Study on Amyotrophic Lateral Sclerosis Patients Using Optical Coherence Tomography and Magnetic Resonance Imaging*. Dissertation. Universität Ulm; 2020. doi:10.18725/OPARU-33085

80. Neeraja K, Nalini A, Preethish-Kumar V, et al. S160. Retinal involvement by optical coherence tomography and its correlation with disease severity in amyotrophic lateral sclerosis. *Clin Neurophysiol*. 2018;129:e201. doi:10.1016/j.clinph.2018.04.520

81. Cennamo G, Montorio D, Ausiello FP, et al. Correlation between Retinal Vascularization and Disease Aggressiveness in Amyotrophic Lateral Sclerosis. *Biomedicines*. 2022;10(10):2390. doi:10.3390/biomedicines10102390

82. Ringelstein M, Albrecht P, Südmeyer M, et al. Subtle retinal pathology in amyotrophic lateral sclerosis. *Ann Clin Transl Neurol*. 2014;1(4):290-297. doi:10.1002/acn3.46

83. Roth NM, Saidha S, Zimmermann H, et al. Optical coherence tomography does not support optic nerve involvement in amyotrophic lateral sclerosis. *Eur J Neurol*. 2013;20(8):1170-1176. doi:10.1111/ene.12146

84. Simonett JM, Huang R, Siddique N, et al. Macular sub-layer thinning and association with pulmonary function tests in Amyotrophic Lateral Sclerosis. *Sci Rep*. 2016;6:29187. doi:10.1038/srep29187

85. Hua R, Xiong J, Li G. Development and validation of a deep learning algorithm based on fundus photographs for estimating the CAIDE dementia risk score. *Publ Online January*. 2022;2. doi:10.1101/2021.08.17.21262156

86. Ferrari L, Huang SC, Magnani G, Ambrosi A, Comi G, Leocani L. Optical Coherence Tomography Reveals Retinal Neuroaxonal Thinning in Frontotemporal Dementia as in Alzheimer’s Disease. *J Alzheimers JAD*. 2017;56(3):1101-1107. doi:10.3233/JAD-160886

87. Kersten HM, Danesh-meyer HV, Kilfoyle DH, Roxburgh RH. Optical coherence tomography findings in Huntington’s disease: a potential biomarker of disease progression. *J Neurol*. 2015;262(11):2457-2465. doi:10.1007/s00415-015-7869-2

88. Amini E, Moghaddasi M, Habibi SAH, et al. Huntington’s disease and neurovascular structure of retina. *Neurol Sci Off J Ital Neurol Soc Ital Soc Clin Neurophysiol*. 2022;43(10):5933-5941. doi:10.1007/s10072-022-06232-3

89. Andrade C, Beato J, Monteiro A, et al. Spectral-Domain Optical Coherence Tomography as a Potential Biomarker in Huntington’s Disease. *Mov Disord Off J Mov Disord Soc*. 2016;31(3):377-383. doi:10.1002/mds.26486

90. Gatto E, Parisi V, Persi G, et al. Optical coherence tomography (OCT) study in Argentinean Huntington’s disease patients. *Int J Neurosci*. 2018;128(12):1157-1162. doi:10.1080/00207454.2018.1489807

91. Di Maio LG, Montorio D, Peluso S, et al. Optical coherence tomography angiography findings in Huntington’s disease. *Neurol Sci Off J Ital Neurol Soc Ital Soc Clin Neurophysiol*. 2021;42(3):995-1001. doi:10.1007/s10072-020-04611-2

92. Gulmez Sevim D, Unlu M, Gultekin M, Karaca C. Retinal single-layer analysis with optical coherence tomography shows inner retinal layer thinning in Huntington’s disease as a potential biomarker. *Int Ophthalmol*. 2019;39(3):611-621. doi:10.1007/s10792-018-0857-7

93. Schmid RD, Remlinger J, Abegg M, et al. No optical coherence tomography changes in premanifest Huntington’s disease mutation carriers far from disease onset. *Brain Behav*. 2022;12(6):e2592. doi:10.1002/brb3.2592

94. Mazur-Michałek I, Kowalska K, Zielonka D, et al. Structural Abnormalities of the Optic Nerve and Retina in Huntington’s Disease Pre-Clinical and Clinical Settings. *Int J Mol Sci*. 2022;23(10):5450. doi:10.3390/ijms23105450

95. Zhang Q, Li J, Bian M, et al. Retinal Imaging Techniques Based on Machine Learning Models in Recognition and Prediction of Mild Cognitive Impairment. *Neuropsychiatr Dis Treat*. 2021;Volume 17:3267-3281. doi:10.2147/NDT.S333833

96. Garcia-Martin E, Rodriguez-Mena D, Satue M, et al. Electrophysiology and Optical Coherence Tomography to Evaluate Parkinson Disease Severity. *Invest Ophthalmol Vis Sci*. 2014;55(2):696-705. doi:10.1167/iovs.13-13062

97. Jiménez B, Ascaso FJ, Cristóbal JA, López del Val J. Development of a prediction formula of Parkinson disease severity by optical coherence tomography. *Mov Disord*. 2014;29(1):68-74. doi:10.1002/mds.25747

98. Satue M, Obis J, Alarcia R, et al. Retinal and Choroidal Changes in Patients with Parkinson’s Disease Detected by Swept-Source Optical Coherence Tomography. *Curr Eye Res*. 2018;43(1):109-115. doi:10.1080/02713683.2017.1370116

99. Shi C, Chen Y, Kwapong WR, et al. CHARACTERIZATION BY FRACTAL DIMENSION ANALYSIS OF THE RETINAL CAPILLARY NETWORK IN PARKINSON DISEASE. *Retina Phila Pa*. 2020;40(8):1483-1491. doi:10.1097/IAE.0000000000002641

100. Xu B, Wang X, Guo J, et al. Retinal Microvascular Density Was Associated With the Clinical Progression of Parkinson’s Disease. *Front Aging Neurosci*. 2022;14:818597. doi:10.3389/fnagi.2022.818597

101. Kwapong WR, Ye H, Peng C, et al. Retinal Microvascular Impairment in the Early Stages of Parkinson’s Disease. *Invest Ophthalmol Vis Sci*. 2018;59(10):4115-4122. doi:10.1167/iovs.17-23230

102. Zhou M, Wu L, Hu Q, et al. Visual Impairments Are Associated With Retinal Microvascular Density in Patients With Parkinson’s Disease. *Front Neurosci*. 2021;15:718820. doi:10.3389/fnins.2021.718820

103. Zhang Y, Zhang D, Gao Y, et al. Retinal Flow Density Changes in Early-stage Parkinson’s Disease Investigated by Swept-Source Optical Coherence Tomography Angiography. *Curr Eye Res*. 2021;46(12):1886-1891. doi:10.1080/02713683.2021.1933054

104. Young JB, Godara P, Williams V, et al. Assessing Retinal Structure in Patients with Parkinson’s Disease. *J Neurol Neurophysiol*. 2019;10(1):485. doi:10.4172/2155-9562.1000485

105. Hajee ME, March WF, Lazzaro DR, et al. Inner Retinal Layer Thinning in Parkinson Disease. *Arch Ophthalmol*. 2009;127(6):737-741. doi:10.1001/archophthalmol.2009.106

106. Albrecht P, Müller AK, Südmeyer M, et al. Optical Coherence Tomography in Parkinsonian Syndromes. Paul F, ed. *PLoS ONE*. 2012;7(4):e34891. doi:10.1371/journal.pone.0034891

107. Satue M, Garcia-Martin E, Fuertes I, et al. Use of Fourier-domain OCT to detect retinal nerve fiber layer degeneration in Parkinson’s disease patients. *Eye*. 2013;27(4):507-514. doi:10.1038/eye.2013.4

108. Rohani M, Langroodi AS, Ghourchian S, Falavarjani KG, SoUdi R, Shahidi G. Retinal nerve changes in patients with tremor dominant and akinetic rigid Parkinson’s disease. *Neurol Sci*. 2013;34(5):689-693. doi:10.1007/s10072-012-1125-7

109. Kirbas S, Turkyilmaz K, Tufekci A, Durmus M. Retinal Nerve Fiber Layer Thickness in Parkinson Disease. *J Neuroophthalmol*. 2013;33(1):62. doi:10.1097/WNO.0b013e3182701745

110. Sen A, Tugcu B, Coskun C, Ekinci C, Nacaroglu SA. Effects of Levodopa on Retina in Parkinson Disease. *Eur J Ophthalmol*. 2014;24(1):114-119. doi:10.5301/ejo.5000338

111. Garcia-Martin E, Larrosa JM, Polo V, et al. Distribution of Retinal Layer Atrophy in Patients With Parkinson Disease and Association With Disease Severity and Duration. *Am J Ophthalmol*. 2014;157(2):470-478.e2. doi:10.1016/j.ajo.2013.09.028

112. Garcia-Martin E, Satue M, Otin S, et al. RETINA MEASUREMENTS FOR DIAGNOSIS OF PARKINSON DISEASE. *RETINA*. 2014;34(5):971. doi:10.1097/IAE.0000000000000028

113. Sari ES, Koc R, Yazici A, Sahin G, Ermis SS. Ganglion Cell–Inner Plexiform Layer Thickness in Patients With Parkinson Disease and Association With Disease Severity and Duration. *J Neuroophthalmol*. 2015;35(2):117. doi:10.1097/WNO.0000000000000203

114. Bayhan HA, Aslan Bayhan S, Tanık N, Gürdal C. The Association of Spectral-Domain Optical Coherence Tomography Determined Ganglion Cell Complex Parameters and Disease Severity in Parkinson’s Disease. *Curr Eye Res*. 2014;39(11):1117-1122. doi:10.3109/02713683.2014.894080

115. Lee JY, Kim JM, Ahn J, Kim HJ, Jeon BS, Kim TW. Retinal nerve fiber layer thickness and visual hallucinations in Parkinson’s Disease. *Mov Disord*. 2014;29(1):61-67. doi:10.1002/mds.25543

116. Quagliato LB, Domingues C, Quagliato EMAB, Abreu EB de, Kara-Junior N. Applications of visual evoked potentials and Fourier-domain optical coherence tomography in Parkinson’s disease: a controlled study. *Arq Bras Oftalmol*. 2014;77:238-242. doi:10.5935/0004-2749.20140061

117. Satue M, Seral M, Otin S, et al. Retinal thinning and correlation with functional disability in patients with Parkinson’s disease. *Br J Ophthalmol*. 2014;98(3):350-355. doi:10.1136/bjophthalmol-2013-304152

118. Schneider M, Müller HP, Lauda F, et al. Retinal single-layer analysis in Parkinsonian syndromes: an optical coherence tomography study. *J Neural Transm*. 2014;121(1):41-47. doi:10.1007/s00702-013-1072-3

119. Roth NM, Saidha S, Zimmermann H, et al. Photoreceptor layer thinning in idiopathic Parkinson’s disease. *Mov Disord*. 2014;29(9):1163-1170. doi:10.1002/mds.25896

120. Stemplewitz B, Keserü M, Bittersohl D, et al. Scanning laser polarimetry and spectral domain optical coherence tomography for the detection of retinal changes in Parkinson’s disease. *Acta Ophthalmol (Copenh)*. 2015;93(8):e672-e677. doi:10.1111/aos.12764

121. Bittersohl D, Stemplewitz B, Keserü M, Buhmann C, Richard G, Hassenstein A. Detection of retinal changes in idiopathic Parkinson’s disease using high-resolution optical coherence tomography and heidelberg retina tomography. *Acta Ophthalmol (Copenh)*. 2015;93(7):e578-e584. doi:10.1111/aos.12757

122. Kaur M, Saxena R, Singh D, Behari M, Sharma P, Menon V. Correlation Between Structural and Functional Retinal Changes in Parkinson Disease. *J Neuroophthalmol*. 2015;35(3):254. doi:10.1097/WNO.0000000000000240

123. Mailankody P, Battu R, Khanna A, Lenka A, Yadav R, Pal PK. Optical coherence tomography as a tool to evaluate retinal changes in Parkinson’s disease. *Parkinsonism Relat Disord*. 2015;21(10):1164-1169. doi:10.1016/j.parkreldis.2015.08.002

124. Pilat A, McLean RJ, Proudlock FA, et al. In Vivo Morphology of the Optic Nerve and Retina in Patients With Parkinson’s Disease. *Invest Ophthalmol Vis Sci*. 2016;57(10):4420-4427. doi:10.1167/iovs.16-20020

125. Eraslan M, Balci SY, Cerman E, Temel A, Suer D, Elmaci NT. Comparison of Optical Coherence Tomography Findings in Patients With Primary Open-angle Glaucoma and Parkinson Disease. *J Glaucoma*. 2016;25(7):e639. doi:10.1097/IJG.0000000000000239

126. Ucak T, Alagoz A, Cakir B, Celik E, Bozkurt E, Alagoz G. Analysis of the retinal nerve fiber and ganglion cell – Inner plexiform layer by optical coherence tomography in Parkinson’s patients. *Parkinsonism Relat Disord*. 2016;31:59-64. doi:10.1016/j.parkreldis.2016.07.004

127. Polo V, Satue M, Rodrigo MJ, et al. Visual dysfunction and its correlation with retinal changes in patients with Parkinson’s disease: an observational cross-sectional study. *BMJ Open*. 2016;6(5):e009658. doi:10.1136/bmjopen-2015-009658

128. Satue M, Rodrigo MJ, Obis J, et al. Evaluation of Progressive Visual Dysfunction and Retinal Degeneration in Patients With Parkinson’s Disease. *Invest Ophthalmol Vis Sci*. 2017;58(2):1151-1157. doi:10.1167/iovs.16-20460

129. Gulmez Sevim D, Unlu M, Gultekin M, Karaca C, Mirza M, Mirza GE. Evaluation of Retinal Changes in Progressive Supranuclear Palsy and Parkinson Disease. *J Neuroophthalmol*. 2018;38(2):151. doi:10.1097/WNO.0000000000000591

130. Gulmez Sevim D, Unlu M, Sonmez S, Gultekin M, Karaca C, Ozturk Oner A. Retinal vessel diameter obtained by optical coherence tomography is spared in Parkinson’s disease. *Int Ophthalmol*. 2019;39(4):813-819. doi:10.1007/s10792-018-0873-7

131. Visser F, Vermeer KA, Ghafaryasl B, et al. In vivo exploration of retinal nerve fiber layer morphology in Parkinson’s disease patients. *J Neural Transm*. 2018;125(6):931-936. doi:10.1007/s00702-018-1872-6

132. Huang J, Li Y, Xiao J, et al. Combination of Multifocal Electroretinogram and Spectral-Domain OCT Can Increase Diagnostic Efficacy of Parkinson’s Disease. *Park Dis*. 2018;2018:e4163239. doi:10.1155/2018/4163239

133. Matlach J, Wagner M, Malzahn U, et al. Retinal changes in Parkinson’s disease and glaucoma. *Parkinsonism Relat Disord*. 2018;56:41-46. doi:10.1016/j.parkreldis.2018.06.016

134. Ma LJ, Xu LL, Mao C jie, et al. Progressive Changes in the Retinal Structure of Patients with Parkinson’s Disease. *J Park Dis*. 2018;8(1):85-92. doi:10.3233/JPD-171184

135. Moschos MM, Chatziralli IP. Evaluation of Choroidal and Retinal Thickness Changes in Parkinson’s Disease Using Spectral Domain Optical Coherence Tomography. *Semin Ophthalmol*. 2018;33(4):494-497. doi:10.1080/08820538.2017.1307423

136. Unlu M, Gulmez Sevim D, Gultekin M, Karaca C. Correlations among multifocal electroretinography and optical coherence tomography findings in patients with Parkinson’s disease. *Neurol Sci*. 2018;39(3):533-541. doi:10.1007/s10072-018-3244-2

137. Aydin TS, Umit D, Nur OM, et al. Optical coherence tomography findings in Parkinson’s disease. *Kaohsiung J Med Sci*. 2018;34(3):166-171. doi:10.1016/j.kjms.2017.11.006

138. Tugcu B, Melikov A, Yildiz GB, et al. Evaluation of retinal alterations in Parkinson disease and tremor diseases. *Acta Neurol Belg*. 2020;120(1):107-113. doi:10.1007/s13760-019-01228-x

139. Tsironi EE, Dastiridou A, Katsanos A, et al. Perimetric and retinal nerve fiber layer findings in patients with Parkinson’s disease. *BMC Ophthalmol*. 2012;12:54. doi:10.1186/1471-2415-12-54

140. Alkabie S, Lange A, Manogaran P, Stoessl AJ, Costello F, Barton JJS. Optical coherence tomography of patients with Parkinson’s disease and progressive supranuclear palsy. *Clin Neurol Neurosurg*. 2020;189:105635. doi:10.1016/j.clineuro.2019.105635

141. Archibald NK, Clarke MP, Mosimann UP, Burn DJ. Retinal thickness in Parkinson’s disease. *Parkinsonism Relat Disord*. 2011;17(6):431-436. doi:10.1016/j.parkreldis.2011.03.004

142. Zou J, Liu K, Li F, Xu Y, Shen L, Xu H. Combination of optical coherence tomography (OCT) and OCT angiography increases diagnostic efficacy of Parkinson’s disease. *Quant Imaging Med Surg*. 2020;10(10):1930-1939. doi:10.21037/qims-20-460

143. Robbins CB, Thompson AC, Bhullar PK, et al. Characterization of Retinal Microvascular and Choroidal Structural Changes in Parkinson Disease. *JAMA Ophthalmol*. 2021;139(2):182-188. doi:10.1001/jamaophthalmol.2020.5730

144. Murueta-Goyena A, Del Pino R, Galdós M, et al. Retinal Thickness Predicts the Risk of Cognitive Decline in Parkinson Disease. *Ann Neurol*. 2021;89(1):165-176. doi:10.1002/ana.25944

145. Berkowitz S, Patel S. Pilot study for neurological and retinal imaging as biomarkers for Parkinson’s disease using optical coherence tomography-angiography. *Invest Ophthalmol Vis Sci*. 2020;61(7):4831.

146. Rascunà C, Russo A, Terravecchia C, et al. Retinal Thickness and Microvascular Pattern in Early Parkinson’s Disease. *Front Neurol*. 2020;11:533375. doi:10.3389/fneur.2020.533375

147. Nunes A, Silva G, Duque C, et al. Retinal texture biomarkers may help to discriminate between Alzheimer’s, Parkinson’s, and healthy controls. *PLoS ONE*. 2019;14(6):e0218826. doi:10.1371/journal.pone.0218826

148. Pillai JA, Bermel R, Bonner-Jackson A. Retinal Nerve Fiber Layer Thinning in Alzheimer’s Disease: A Case-Control Study in Comparison to Normal Aging, Parkinson’s Disease, and Non-Alzheimer’s Dementia. *Am J Alzheimers Demen*. 2016;31(5):430-436. doi:10.1177/1533317515628053

149. O’Bryhim BE, Apte RS, Kung N, Coble D, Stavern GP. Association of Preclinical Alzheimer Disease With Optical Coherence Tomographic Angiography Findings. *JAMA Ophthalmol*. 2018;136(11):1242-1248. doi:10.1001/jamaophthalmol.2018.3556

150. Snyder PJ, Johnson LN, Lim YY, et al. Nonvascular retinal imaging markers of preclinical Alzheimer’s disease. *Alzheimers Dement Diagn Assess Dis Monit*. 2016;4:169-178. doi:10.1016/j.dadm.2016.09.001

151. Asanad S, Fantini M, Sultan W. Retinal nerve fiber layer thickness predicts CSF amyloid/tau before cognitive decline. *PLoS ONE*. 2020;15(5). doi:10.1371/journal.pone.0232785

152. Zee B, Wong Y, Lee J, et al. Machine-learning method for localization of cerebral white matter hyperintensities in healthy adults based on retinal images. *Brain Commun*. 2021;3(3):fcab124. doi:10.1093/braincomms/fcab124
