# Supplementary material for: Deep Learning and Machine Learning Algorithms for Retinal Image Analysis in Neurodegenerative Disease: Systematic Review of Datasets and Models
Source: Transl Vis Sci Technol. 2024 Feb 21;13(2):16. doi: 10.1167/tvst.13.2.16 (PMC10893898; doi:10.1167/tvst.13.2.16)
Supplement: Supplement 2 [file tvst-13-2-16_s002.docx]

**Supplementary Table 2** - Machine learning models for retinal image analysis in neurodegenerative disease with additional information

| Disease | Model task | Model Metrics | Dataset Information | Input Data | Data Augmentation, Processing, or Feature Extraction | General Architecture | Other Elements | Attention Map or Physiologic Correlates | Limitations | Ref |
| --- | --- | --- | --- | --- | --- | --- | --- | --- | --- | --- |
| AD | Classification: clinical AD or controls using color fundus images | Binary Accuracy  82.44% | 122 AD images  122 ctrl images  80/20% dev/test partition | Vessel Map output from UNet | Image quality selector (a classifier)  UNet (a CNN) for vessel Segmentation | multi-modal pipeline with each step trained separately: image quality selector, UNet for vessel segmentation, SVM classifier | multi-modal pipeline with each step trained separately: image quality selector, UNet for vessel segmentation, SVM classifier | saliency map generation via occlusion tests highlighted small vessels and capillaries | small sample size  Dataset known to have healthier population compared with the general population  Substantial amount of AD may have been undocumented, including some of the controls | ^82^ |
| AD | Classification: Clinical AD or controls using multimodal imaging (OCT, OCTA, color and autofluorescence fundus photos) and also patient metadata | AUC using all inputs together 0.836 (CI 0.729, 0.943)  AUC using GC-IPL maps, quantitative OCT data and patient data: AUC 0.841 (95% CI 0.739, 0.943)  AUC for GCL-IPL maps as only input:  0.809 (95% CI 0.700, 0.919) | 222 eyes from 123 healthy patients  62 eyes from 36 AD patients  approx. 65/10/25%  train/dev/test partition | GC-IPL thickness maps, SCP-OCTA images, UWF color and autofluorescence fundus images, OCT and OCTA quantitative data,  metadata | downsizing,  rotating, shifting, cropping, zooming  Feature maps were generated for each imaging modality using a CNN with the first 5 layers of pretrained Res-Net 18. | Each feature map, and also quantitative imaging data plus patient metadata, were passed to respective FC layers, which then converged in one final output layer.  Apart from the multimodal model, separate models were also constructed separately for each input image modality. | UNet for Lash and lid artifact removal during preprocessing was attempted but had no effect on model performance | OCT: attention on foveal avascular zone  OCTA: scattered foci of attention corresponding to areas with decreased capillary density  Fundus image: inconsistent, multifocal areas of attention  GC-IPL maps were the most useful single inputs for prediction | use of cropped images may have deprived the model of potentially pertinent information in the peripheral retina  shallow model due to small dataset limits complexity of feature detection  AD patients were diagnosed clinically rather than imaging or ApoE4 genetic basis | ^62^ |
| AD | Classification:  FBB-PET (+) or (-) negative at baseline and after 24 month period predicted using baseline right eye OCT images in patients with subjective cognitive decline (SCD) | Logistic Classifier accuracy not reported  Multivariate regression showed 8% and 6% higher probability of PET+ at baseline and 2 years, respectively, per 1 μm of increased inner nasal macular thickness. | Baseline: 114 FBB-PET(-), 15 FBB-PET(+)  at 2 years:  107 sustained FBB-PET(-), 22 FBB-PET(+) | 3 OCT modalities: peripapillary RNFL, macular ETDRS, macular multilayer. | automated OCT layer segmentation to determine thicknesses of 16 retinal regions | 16 univariate logistic regression models to predict baseline PET status  16 univariate logistic regression models to predict amyloid PET positivity after 24 months | multivariate logistic regression model using nasal macular thickness and also patient metadata (education, gender, age, APOE ε4 status and OCT retinal image quality) | independent p-values by T-test showed all five ETDRS inner macular subfields (superior, center, inferior, nasal, temporal) were significantly thicker in new-onset FBB-PET positive individuals compared with controls. | relatively small sample size (especially for the subgroup with abnormal Aβ)  short follow-up period relative to AD time course | ^83^ |
| AD | Classification: clinical AD or control from bilateral retinal fundus photographs and metadata  Classification of FBB-PET (+) or (-) from bilateral retinal fundus photographs and metadata | Best AUC 0.91 (95% CI 0.81-1.0)  Best AUC 0.86 (95% CI 0.7-1.0) | 12,949 images from 648 AD patients and 3240 controls | four retinal photographs (optic nerve head-centered and macula- centered fundus images from both eyes) | cropping, background subtraction, rim removal, channel separation  Feature extraction using Efficient Net-b2 | Feature fusion via concatenation of extracted features from the four images followed by an FC layer, followed by fusion of demographic features by deep bilinear transformation, followed by FC output layer. | Domain-specific batch normalization in convolution blocks for unsupervised domain adaptation to address dataset discrepancy | Grad-CAM maps show attention to inferior arcade and inferior periphery   Performance of the bilateral model was better than that of the unilateral model in the testing | still relatively small dataset (<1000 AD patients)  labeling based on clinical diagnosis  substantial overlap between Alzheimer's disease and cerebrovascular disease (shared risk factors) | ^61^ |
| AD | Classification: Clinical Alzheimer disease or controls using OCT RNFL images | 99% sensitivity and specificity  Per-pixel 99.65% binary accuracy for OCT segmentation | 25 AD  25 Ctrls,  one image per eye (total of 100 images). | OCT RNFL | noise extraction using median filter, RGB normalization, wavelon scale and shift parameters, downscaling  Artificial fixed-grid wavelet network for segmentation for feature extraction from segmented image | radial basis function-based neural network classifier | Wavelet networks:   Extracted features included ellipticity  thickness ratio  variance of curvature  regional minima  area  perimeter  2nd, 3rd, fourth moments | - | small dataset  wavelet networks are not well-described in the literature  stage of AD and diagnostic criteria unclear | ^84^ |
| AD | Classification: AD or controls using combination of snapshot hyperspectral retinal imaging and OCT | best AUC of 0.79 using the I2 region of interest (see publication for how region maps on the fundus) | 17 AD  22 controls | snapshot hyperspectral retinal imaging and OCT RNFL | snapshot hyperspectral retinal imaging of 4 different regions of interest, and RNFL thickness and vertical cup-to-disc ratio (vCDR) measurements | 8 different linear discriminant classifiers trained independently using normalized hyperspectral data from 4 fundus regions of interest and either fused or not fused with OCT RNFL thickness measurement data. | - | largest hyperspectral retinal imaging differences were observed in the superior S1 region, however,  I2 region was the most informative for discriminating between AD and controls | lack of biomarker confirmation of AD in 10/17 subjects  snapshot imaging obtaining spatial and spectral imaging at once sacrifices the resolution of both modalities | ^85^ |
| AD | Classification: AD or control using Fourier-domain OCT RNFL measurements | best AUC of 0.965 for validation set | training set: 151 advanced AD patients  61 controls  validation set: 50 healthy eyes and 50 AD eyes | RNFL OCT | 768 RNFL measurements at distinct points on the retina | linear discriminant classifier | - | - | Inclusion criteria of advanced AD was used to enhance the likelihood of OCT measurements being diagnostically useful, but limits the generalizability of the model | ^86^ |
| MCI | Classification: Mild Cognitive Impairment (MCI), dementia, controls using fundus photos. | AUC for MCI prediction on validation set: 0.87  AUC for dementia prediction on validation set: 0.86 | total 332 images from the following patient groups:  38 controls  26 MCI  22 dementia  ~70/30% train/validation split | RGB fundus photo | random translation, flipping, enlargement, miscut, and filling, and normalization | histogram of oriented gradient (HOG) was used for feature extraction  SVM classifier | ELM classifier was also trained independently but did not perform as well as the SVM model. | Other SVMs models were trained on segmentation maps only which were generated from the raw fundus photo. These models had poorer performance compared with models that used the raw fundus image only. | single-center data lacking external validation  cross-sectional rather than longitudinal data  Treats different subtypes of the stroke equally although various types may lead to different degrees and domains of cognitive impairment | ^87^ |
| MCI, AD | Segmentation: mapping retinal vessels in OCTA images | per-pixel AUC of 0.95 for superficial vascular complex segmentation  per-pixel AUC of 0.97 for deep vascular complex segmentation | ROSE-1 dataset: 117 OCTA images from 29 AD patients and 13 controls | OCTA image | random rotation from −10° to 10° | Overall, a coarse stage followed by a fine stage. Throughout both stages, pixel-level and centerline-level vessel segmentation branches run in parallel. The output layer fuses the pixel-level and centerline-level branches. | Coarse stage: Composed of ResNeSt blocks and split attention module.  Fine stage: mini 3-layer CNN | Novel split-based coarse-to-fine vessel segmentation network for blood vessel segmentation in OCTA, aimed at detecting thick and thin vessels separately | AD diagnosis was based on the NINCDS-ADRDA criteria, not amyloid PET or CSF studies  OCTA is known to have projection artifacts | ^88^ |
| PD | Regression: retinal age gap (predicted - actual age) and demographic covariates for 5-year incident PD risk prediction | AUC 0.708 for 5-year incident PD risk prediction | 35,917 participants with no PD at baseline | Bilateral color fundus photos and patient metadata | CNN used to predict retinal age. Predicted - actual age was used as the key input feature in the Cox proportional hazards model. | Cox proportional hazards multivariate regression model using predicted retinal age gap and other patient covariates | - | 10% increase in PD risk per 1-year increase in retinal age gap | UK Biobank underrepresents older, more ill members of the population  limited number of incident PD cases  unable to investigate dynamic changes in retinal age gap and incident PD | ^35^ |
| PD, AD | Classification: AD, PD, or control using macular OCT images | Median sensitivities of 88.7%, 79.5%, and 77.8%, for HC, AD, and PD eyes, respectively. | 20 AD  28 PD  27 Ctrl | Macular OCT images | horizontal flipping of left eye images, intensity correction  Segmentation of macular OCT into retinal layers and generation of mean value fundus (MVF) maps for 6 layers: RNFL, GCL, IPL, INL, OPL, and ONL. | support vector machine (SVM) classifier.  18 independent binary SVM models were developed, one per each of the six retinal layers at study and between any two of the three possible groups at study: the HC, AD, and PD groups. | - | Global texture metrics were used in 66.7% of the classification models suggesting that these differences are spread over the entire macular region.  texture features tended to convey information on differences present in the retina that were not conveyed by thickness features | unclear whether there are retinal layer thickness differences between controls and AD or PD patients; the data features may not provide much signal.  Retinal thickness differences are more likely to be present at advanced stages of disease. | ^75^ |
| Cardiovascular Risk Factors, Aging, and Incidence of Dementia (CAIDE) risk score | Regression: estimate the CAIDE risk score using fundus images  Classification: high dementia risk (CAIDE > 10). | R=0.76 for predicted versus actual CAIDE score in the external validation set  AUC 0.926 (95% CI 0.913 - 0.939) on external validation dataset for identifying individuals with high dementia risk | 271,864 participants (training set)  20,690 participants (validation set) | color fundus photographs taken using various acquisition devices | image normalization, random cropping, rotation (30 deg), horizontal flipping, | InceptionResNetV2 | - | Attention maps in patients with high CAIDE scores highlighted parafoveal vessels.  Maps for patients with low scores focused on the peripheral regions of the vessel arcades. | Educational level and physical inactivity were interpolated from other data (sex, age, BMI).  Significant age difference between the development and external validation datasets  only included Chinese participants, limiting generalizability to other ethnicities. | ^66^ |
| Cognitive Scores | Regression: using retinal fundus photos and metadata to predict cognitive scores in healthy volunteers  Classification: APOE4 gene (+) or (-) using retinal fundus photos and metadata | Fundus images and metadata only explained 22.4% of the variance in cognitive scores in this model.  AUC 0.47 for APOE4 classification | 25,737 fundus images labeled with cognitive scores     26,622 fundus images labeled with APOE4 genotype | Unilateral color fundus image | image quality selector  horizontal and vertical flipping, rotation, color normalization  shearing and affine transformations were omitted | Pretrained network (InceptionV3, MobileNetV2 or EfficientNetB3) extracted image features, concatenated with patient metadata, then passed through two FC layers, the last of which being the output layer. | Exploratory and confirmatory factor analysis  Model validation on other dependent variables (sex, age, BMI) | Saliency maps generated using soft attention highlighted the optic nerve head as the most influential feature for predicting cognitive scores.  Metadata alone explained more sample variance (20.4%) than fundus images alone (9.3%). | The study population underrepresented individuals with severe cognitive impairment (only healthy individuals were included)  study was limited to the Canadian population  Metadata, including sex, was self-reported potentially introducing bias | ^65^ |
| Retinal Age | Regression: predict  age using fundus photographs (retinal age) in healthy aging individuals | R = 0.81 comparing predicted and actual age | 19,200 fundus images of 11052 participants without chronic disease for the age prediction model. | Bilateral color fundus photos | color normalization, data augmentation using horizontal or vertical flips. | Xception convolutional neural network- a 71-layer-deep pretrained network using ImageNet database similar to inception V3 but with depthwise-separable convolutions. | - | attention maps highlighted scattered regions around small retinal vessels in all quadrants  Each 1-year increment in retinal age gap (predicted - actual age) was associated with 2% increase in all-cause mortality risk | cross-sectional nature of the study rather than longitudinal does not allow for investigation of changes in trajectory  UK biobank comprised of more healthy population compared with general population | ^68^ |
| White matter hyperintensities | Detection of white matter hyperintensities with age-related white matter change (ARWMC) score > 2 and classification of brain region to one of 6 potential regions: left and right frontal lobes, parietal–occipital lobes, or basal ganglia using fundus photos | AUC of 0.955 based on 10-fold cross-validation for detection of ARWMC > 1 across any of the 6 brain regions | bilateral images from 240 subjects with ARWMCs in various brain regions  75/25% train/validation split | bilateral RGB fundus photos | no pre-processing specified | parallel convolutional neural network models ResNet50 (for pixel-based feature extraction) and ARIA (for texture, spectral, and fractal feature extraction).  Extracted features passed to Glmnet, then SVM classifier | Classification and regression tree approach for classification problem of localizing white matter hyperintensities | Data from both eyes was important for detecting age-related white matter changes in the brain regions | small sample size  regional WMH load in the study group is not as high as in other patient groups. | ^64^ |

SVM: Support vector machine, CI: 95% confidence interval, mGCIPL: macular ganglion cell layer and inner plexiform layer, pRNFL: peripapillary retinal nerve fiber layer, SVP: superficial venous plexus, AD: Alzheimer’s disease, PD: Parkinson’s disease, ALS: Amyotrophic lateral sclerosis, HD: Huntington’s disease MCI: Mild cognitive impairment, D-US: Dementia, un-specified; ARIA, automatic retinal image analysis. WMH, white matter hyperintensities.
